# Supplementary material for: Engineered Cas12i2 is a versatile high-efficiency platform for therapeutic genome editing
Source: Nat Commun. 2022 May 20;13:2833. doi: 10.1038/s41467-022-30465-7 (PMC9122993; doi:10.1038/s41467-022-30465-7)
Supplement: Supplementary file 2 — Supplementary Information [file 41467_2022_30465_MOESM2_ESM.pdf]

Engineered Cas12i2 is a versatile high-efficiency platform for therapeutic genome editing

McGaw, C., et al.

Supplementary Materials and Tables

Supplementary Methods

In vitro expression of SpCas9 and *in vitro* cleavage assay

The reconstituted cell-free protein synthesis system was manufactured at Arbor Biotechnologies using protocols derived from previous publications[1, 2]. The cell-free protein synthesis reagents were used in 25 μL reactions containing 150 ng of the linear DNA template encoding SpCas9, 40 units murine RNAase inhibitor (40 U/μL), 2 nM *in vitro* transcribed target and non-target gRNA, and 150 ng GFP target DNA (sequences in Supplementary Table 2). The reactions were incubated at 37°C and aliquots (2.5 μL) were taken at different time points for both protein expression and DNA cleavage analysis. For protein expression analysis, aliquots were mixed with 7.5 water and 2.5 μL NuPAGE LDS Sample Buffer (Thermo Fisher #NP0007) supplemented with 10 mM DTT. After heating at 95°C for 2 minutes, the samples were analyzed on 4-12% Bis-Tris NuPAGE gel (Thermo Scientific #NP0321BOX) (Supplementary Fig. 3a). For *in vitro* DNA cleavage analysis, aliquots were mixed with 7.5 water and treated with RNase Cocktail Enzyme mix and Proteinase K (Thermo Scientific #EO0491) at 37°C for 20 min. The samples were analyzed on 6% TBE gel (Thermo Scientific EC6265BOX) (Supplementary Fig. 3b).

Description of the *in vitro* fluorescent reporter assay

The fluorescent reporter assay was designed to couple the effector and gRNA synthesis with a fluorescent reporter-based activity assay (Supplementary Fig. 4a). The CRISPR-Cas effectors (Cas12i and SpCas9), pre-crRNA or gRNAs, and *E. coli* transcription factor sigma factor 28 (σ^{28}) were expressed under the T7 promoter (P_{T7}), whereas green and red fluorescent protein (GFP and RFP) reporters were expressed under the control of a σ^{28}-dependent promoter (fliC). Pre-crRNAs and gRNAs were designed to target GFP, but not RFP. The RFP served as an internal control for gene expression and fluorescence measurement. The fluorescence of the reaction mixture was measured over a course of 12 hrs.

This assay is a mix-and-read one-pot reaction in which DNA templates are all added at the beginning to initiate *in vitro* transcription and protein synthesis. As the *in vitro* reaction starts, the effector and gRNA are produced immediately due to the strong T7 promoter, whereas GFP and RFP (under fliC promoter) are not produced until enough σ^{28} protein is made. The delay in GFP expression provides time for newly synthesized effector and gRNA to form a ribonucleoprotein complex (RNP) that cleaves the GFP DNA template. DNA cleavage in the GFP-coding region abolishes or decreases GFP production, resulting in a depletion of GFP fluorescence compared to non-target gRNA that targets a site outside of the GFP gene. RFP, on the other hand, is expressed in the same reaction without the RNP interference, generating RFP protein as a control for normalizing GFP signals (Supplementary Fig. 4a).

Validation of the *in vitro* fluorescence reporter assay with SpCas9 and Cas12i2 WT

SpCas9 was chosen as both a control and a benchmark to validate the assay (Supplementary Fig. 4b-d). Initially, we set up *in vitro* reactions expressing SpCas9, non-target gRNA, and gRNAs targeting two GFP sites (target 9 and 10). GFP and RFP fluorescence was measured every 10 min at 37°C for 18 hr. Both GFP-targeting gRNAs almost completely abolished GFP fluorescence compared to the non-target gRNA control (Supplementary Fig. 4b, target 9 and 10), suggesting that *in vitro* synthesized SpCas9 efficiently cleaved the GFP template and prevented GFP expression. RFP fluorescence, on the other hand, remained relatively unchanged between targets and non-target, as the RFP template was not the target for SpCas9 (Supplemental Fig. 4c). The small variations of target and non-target RFP fluorescence were likely caused by variations in gene expression and fluorescence measurement, which was accounted for by normalizing GFP with RFP fluorescence. Depletion values, calculated as the ratios of normalized non-target gRNA fluorescence over normalized target gRNA fluorescence, was plotted to show that GFP fluorescence was efficiently depleted by SpCas9 over 20-fold for both targets after ~ 5 hr (Supplemental Fig. 4d).

Similar reactions were performed with Cas12i2 WT on two GFP sites showing GFP fluorescence was depleted only ~2 fold after ~ 5 hr for both targets (Supplementary Fig.4 e-g). The low depletion of Cas12i2 was due to substantial GFP fluorescence still remaining in the presence of GFP-targeting gRNAs (Supplementary Fig. 4e), indicating that *in vitro* synthesized Cas12i2 WT in these experiments failed to completely cleave the GFP template to prevent GFP expression.

To expand the assay with more targets, we showed that expression of SpCas9 and gRNAs targeting 10 targets in the GFP gene resulted in a decrease of GFP signals by

an average of 27.7-fold (Supplementary Fig. 5). In comparison, Cas12i2 WT decreased GFP signals by only 1.9-fold across 20 targets (Supplementary Fig. 5). These data are consistent with the low editing efficiency observed for Cas12i2 WT compared to SpCas9 in HEK293T cells (Fig. 1a). Though the *in vitro* fluorescence reporter assay measures both cell-free expression and *in vitro* cleavage activity, we hypothesized that the fold-decrease in GFP signal (depletion) of Cas12i2 WT correlates with the intrinsic DNA targeting activity of the effector, and therefore, could be used to screen mutations with improved dsDNA cleavage activity, which might translate to enhanced editing activity in mammalian cells.

In silico off-target prediction and off-target analysis

For each target, potential off-target sites within an edit distance of 4 were identified by searching for genomic sequences adjacent to the PAM (ABR001: TTN, spCas9: NRG), the Levenshtein distance between the target sequence and these PAM-adjacent sequences was calculated, and sequences with an edit distance greater than 4 were removed. The top predicted off-targets were selected by sorting the off-targets by edit distance and the number of mismatches. Primer pairs for each off-target site were generated by centering the off-target sequence within a 120-140 base pair amplicon, and targeted amplicon sequencing was performed using these primers. The sequencing data was analyzed using the indel analysis pipeline as described above, with an additional step to correct for background noise using the indel rate of paired negative control samples as a prior in maximum likelihood estimation (MLE).

Supplementary Table 1. Comparison of Cas12i with type II Cas9 and other type V Cas12 systems

Ortholog family	effector	Size (aa)	pre-crRNA processing	Tracr RNA	PAM	dsDNA cleavage	ssDNA cleavage
Type II, Cas9[3]	SpCas9	1368	no	yes	3'NGG	Blunt cut	no
Type V-A, Cas12a[4]	AsCpf1	1307	yes	no	5'TTTV	5' overhang	yes
Type V-B, Cas12b[5]	BhCas 12b	1108	no	yes	5'ATTN	5' overhang	yes
Type V-E, Cas12e[6] (CasX)	DpbCasX	986	no	yes	5'TTCN	5' overhang	minimal
Type V-F Cas12f (Cas14)[7, 8, 9]	UniCas 12f	529	no	yes	5'TTTN	yes	yes
Type V-J (Cas12j)[10, 11]	Cas phi	700-800	yes	no	5' T-rich	yes	yes
Type V-I, Cas12i[12]	Cas12i1	1093	yes	no	5'TTN	yes	yes
	Cas12i2	1054	yes	no	5'TTN	yes	yes

Supplementary Table 2. A list of DNA sequences used in fluorescent reporter assay

Name	Sequence
pUCK01_PfliC_mh6_dGFP	ccccttgtattactgtttatgtaagcagacaggatgcgtccggcgtagaggatcgagatctccaaaaaatggctgtttttgaaaaaaattctaaaggttgttttacgacaga<br>cgataacagggttgaaataattttgtttaactttaagaaggagatttaaatatgaaaatcgaagaaggtaaaggtcaccatcaccatcaccacggatccatgacggcat<br>tgacggaaggtgcaaaactgtttgagaaagagatcccgtatatcaccgaactggaaggcgacgtcgaaggtatgaaatttatcattaaaggcgagggtaccggtga<br>cgcgaccacgggtaccattaaagcgaaatacatctgcactacgggcgacctgccggtcccgtgggcaaccctggtgagcaccctgagctacggtgttcagtgtttcg<br>ccaagtacccgagccacatcaaggatttctttaagagcgccatgccggaaggttatacccaagagcgtaccatcagcttcgaaggcgacggcgtgtacaagacgc<br>gtgctatggttacctacgaacgcggttctatctacaatcgtgtcacgctgactggtgagaactttaagaaagacggtcacattctgcgtaagaacgttgcattccaatgcc<br>cgccaagcattctgtatattctgcctgacaccgttaacaatggcatccgcgttgagttcaaccaggcgtacgatattgaaggtgtgaccgaaaaactggttaccaaatgc<br>agccaaatgaatcgtccgttggcgggctccgcggcagtgcatatcccgcgttatcatcacattacctaccacaccaaactgagcaaagaccgcgacgagcgccgtg<br>atcacatgtgtctggtagaggtcgtgaaagcggttgatctggacacgtatcagtaataaaaagcccgaaaggaagctgagttggctgctgccaccgctgagcaataa<br>ctagcataaccccttggggcctctaaacgggtcttgaggggttttttgctgaaaggaggaactatatccggcttcctcgctcactgactcgctgcgctcggtcgttcggctg<br>cggcgagcggtatcagctcactcaaaggcggtaatacggttatccacagaatcaggggataacgcaggaaagaacatgtgagcaaaaggccagcaaaaggcc<br>aggaaccgtaaaaaggccgcgttgctggcgtttttccataggctccgcccccctgacgagcatcacaaaaatcgacgctcaagtcagaggtggcgaaacccgaca<br>ggactataaagataccaggcgtttccccctggaagctccctcgtgcgctctcctgttccgaccctgccgcttaccggatacctgtccgcctttctcccttcgggaagcgtgg<br>cgctttctcatagctcacgctgtaggtatctcagttcggtgtaggtcgttcgctccaagctgggctgtgtgcacgaaccccccgttcagcccgaccgctgcgccttatccgg<br>taactatcgtcttgagtccaacccggtaagacacgacttatcgccactggcagcagccactggtaacaggattagcagagcgaggtatgtaggcggtgctacagagtt<br>cttgaagtggtggcctaactacggctacactagaagaacagtatttggtatctgcgctctgctgaagccagttaccttcggaaaaagagttggtagctcttgatccggca<br>aacaaaccaccgctggtagcggtggtttttttgtttgcaagcagcagattacgcgcagaaaaaaaggatctcaagaagatcctttgatcttttctacggggtctgacgctc<br>agtggaacgaaaactcacgggtggcacttttcggggaaatgtgcgcggaacccctatttgtttatttttctaaatacattcaaatatgtatccgctcatgaattaattcttaga<br>aaaactcatcgagcatcaaatgaaactgcaatttattcatatcaggattatcaataccatatttttgaaaaagccgtttctgtaatgaaggagaaaactcaccgaggcag<br>ttccataggatggcaagatcctggtatcggtctgcgattccgactcgtccaacatcaatacaacctattaatttcccctcgtcaaaaataaggttatcaagtgagaaatca<br>ccatgagtgacgactgaatccggtgagaatggcaaaagtttatgcatttctttccagacttgttcaacaggccagccattacgctcgtcatcaaaatcactcgcatcaac<br>caaaccgttattcattcgtgattgcgcctgagcgagacgaaatacgcgatcgctgttaaaaggacaattacaaacaggaatcgaatgcaaccggcgcaggaacact<br>gccagcgcatcaacaatattttcacctgaatcaggatattcttctaatacctggaatgctgttttcccggggatcgcagtggtgagtaaccatgcatcatcaggagtacgg<br>ataaaatgcttgatggtcggaagaggcataaattccgtcagccagtttagtctgaccatctcatctgtaacatcattggcaacgctacctttgccatgtttcagaaacaact<br>ctggcgcatcgggcttcccatacaatcgatagattgtcgcacctgattgcccgacattatcgcgagcccatttatacccatataaatcagcatccatgttggaatttaatcg<br>cggcctagagcaagacgtttcccgttgaatatggctcataaca
pUCK01_PfliC_mh6_RFP	ccccttgtattactgtttatgtaagcagacaggatgcgtccggcgtagaggatcgagatctccaaaaaatggctgtttttgaaaaaaattctaaaggttgttttacgacaga<br>cgataacagggttgaaataattttgtttaactttaagaaggagatttaaatatgaaaatcgaagaaggtaaaggtcaccatcaccatcaccacggatccatggtcagca<br>agggggaggaagacaatatggctattatcaaggaattcatgcgcttcaaggtgcatatggaaggaagcgtgaatggacacgaattcgagatcgaaggcgagggg<br>gagggtcgcccttatgaaggcacacaaacagctaaactgaaagtgacgaagggagggccgcttcccttcgcttgggacattctttcaccccagttcatgtatggttcaa<br>aggcttatgtcaagcacccggcggacattccagactacttaaaattgtcgttccccgaggggtttaaatgggaacgcgttatgaatttcgaggatggggagtcgtaac<br>ggttacccaggacagtagcctgcaggatggcgagttcatctacaaagtgaaattgcgcgggacgaacttccctagcgatgggccagtcatgcagaagaaaacgat<br>gggatgggaagcgtcatccgagcgcatgtatcctgaagatggtgctttaaaaggtgagatcaagcagcgtttgaaactgaaggacgggggccattatgatgctgaag<br>ttaaaacgacatataaggccaagaagccagttcaactgccaggggcttataatgttaatattaaattagacattacgagccataatgaagattacacgattgtcgagca

	atacgagcgcgcagaaggacgccactcaacgggggcatggacgagctgtacaagtaaaaagcccgaaaggaagctgagttggctgctgccaccgctgagcaataactagcataaccccttggggcctctaaacgggtcttgaggggttttttgctgaaaggaggaactatatccggcttcctcgctcactgactcgctgcgctcggtcgttcggctgcggcgagcggtatcagctcactcaaaggcggtaatacggttatccacagaatcaggggataacgcaggaaagaacatgtgagcaaaaggccagcaaaaggccaggaaccgtaaaaaggccgcgttgctggcgtttttccataggctccgcccccctgacgagcatcacaaaaatcgacgctcaagtcagaggtggcgaaacccgacaggactataaagataccaggcgtttccccctggaagctccctcgtgcgctctcctgttccgaccctgccgcttaccggatacctgtccgcctttctcccttcgggaagcgtggcgctttctcatagctcacgctgtaggtatctcagttcggtgtaggtcgttcgctccaagctgggctgtgtgcacgaaccccccgttcagcccgaccgctgcgccttatccggtaactatcgtcttgagtccaacccggtaagacacgacttatcgccactggcagcagccactggtaacaggattagcagagcgaggtatgtaggcggtgctacagagttcttgaagtggtggcctaactacggctacactagaagaacagtatttggtatctgcgctctgctgaagccagttaccttcggaaaaagagttggtagctcttgatccggcaaacaaaccaccgctggtagcggtggtttttttgtttgcaagcagcagattacgcgcagaaaaaaaggatctcaagaagatcctttgatcttttctacggggtctgacgctcagtggaacgaaaactcacgggtggcacttttcggggaaatgtgcgcggaacccctatttgtttatttttctaaatacattcaaatatgtatccgctcatgaattaattcttagaaaaactcatcgagcatcaaatgaaactgcaatttattcatatcaggattatcaataccatatttttgaaaaagccgtttctgtaatgaaggagaaaactcaccgaggcagttccataggatggcaagatcctggtatcggtctgcgattccgactcgtccaacatcaatacaacctattaatttcccctcgtcaaaaataaggttatcaagtgagaaatcaccatgagtgacgactgaatccggtgagaatggcaaaagtttatgcatttctttccagacttgttcaacaggccagccattacgctcgtcatcaaaatcactcgcatcaaccaaaccgttattcattcgtgattgcgcctgagcgagacgaaatacgcgatcgctgttaaaaggacaattacaaacaggaatcgaatgcaaccggcgcaggaacactgccagcgcatcaacaatattttcacctgaatcaggatattcttctaatacctggaatgctgttttcccggggatcgcagtggtgagtaaccatgcatcatcaggagtacggataaaatgcttgatggtcggaagaggcataaattccgtcagccagtttagtctgaccatctcatctgtaacatcattggcaacgctacctttgccatgtttcagaaacaactctggcgcatcgggcttcccatacaatcgatagattgtcgcacctgattgcccgacattatcgcgagcccatttatacccatataaatcagcatccatgttggaatttaatcgcggcctagagcaagacgtttcccgttgaatatggctcataaca
T7_Sigma28	cccgcgaaattaatacgactcactataggggaattgtgagcggataacaattcccctctagaaaataattttgtttaactttaagaaggagatttaaatatgaaaatcgaagaaggtaaaggtcaccatcaccatcaccacggtggtcatatgaactcgctttatacggctgagggtgtcatggacaagcatagtttgtggcaacgctatgttcctcttgtgcgtcacgaggcgttacgtttacaagtgcgtcttcctgcgagcgtcgaattagatgacttattacaagcgggagggatcgggttattgaatgcagtagaacgctacgacgctcttcaggggacggctttcacgacgtatgcagtccagcgtatccgcggagcaatgttagatgagctgcgttctcgtgattgggtgccgcgcagcgtgcgtcgtaatgctcgtgaggttgctcaagccatcggccagctggaacaagaactgggtcgcaatgccacagagactgaagttgcagaacgcttagggatcgacattgcggattatcgtcaaatgttattggacactaacaattctcagttattctcctacgacgaatggcgtgaagaacacggagactcaatcgagcttgtcactgacgaccaccagcgcgagaatccgttgcagcagttattggactcaaaccttcgtcagcgtgtgatggaagccattgaaaccttgccggaacgtgagaagctggtactgactttatactatcaggaagagttaaatcttaaagagattggtgctgttttggaagttggggagtcacgcgtgtcacaactgcactcacaggccatcaagcgcttgcgcaccaagttagggaaactttaataactgctaacaaagcccgaaaggaagctgagttggctgctgccaccgctgagcaataactagcataaccccttggggcctctaaacgggtcttgaggggttttttgctgaaaggaggaactatatccgg
T7_Cas12i2_Effector_wt	cccgcgaaattaatacgactcactataggggaattgtgagcggataacaattcccctctagatttacactttatgcttccggctcgtatgtttctagaaagcttaaataattttgtttaactttaagaaggagatttaaatatgaaaatcgaagaaggtaaaggtcaccatcaccatcaccacatgagcagcgcgatcaaaagctacaagagcgttctgcgtccgaacgagcgtaagaaccaactgctgaaaagcaccattcagtgcctggaagacggtagcgcgttctttttcaagatgctgcaaggcctgtttggtggcatcaccccggagattgttcgtttcagcaccgaacaggagaaacagcaacaggatatcgcgctgtggtgcgcggttaactggttccgtccggtgagccaagacagcctgacccacaccattgcgagcgataacctggtggagaagtttgaggaatactatggtggcaccgcgagcgacgcgatcaaacagtacttcagcgcgagcattggcgaaagctactattggaacgactgccgtcaacagtactatgatctgtgccgtgagctgggtgttgaggtgagcgacctgacccatgatctggagatcctgtgccgtgaaaagtgcctggcggttgcgaccgagagcaaccagaacaacagcatcattagcgttctgtttggcaccggcgaaaaagaggaccgtagcgtgaaactgcgtatcaccaagaaaattct

	ggaggcgatcagcaacctgaaagaaatcccgaagaacgttgcgccgattcaagagatcattctgaacgtggcgaaagcgaccaaggaaaccttccgtcaggtgt<br>atgcgggtaacctgggtgcgccgagcaccctggagaaatttatcgcgaaggacggccaaaaagagttcgatctgaagaaactgcagaccgacctgaagaaagtt<br>attcgtggtaaaagcaaggagcgtgattggtgctgccaggaagagctgcgtagctacgtggagcaaaacaccatccagtatgacctgtgggcgtggggcgaaatgt<br>tcaacaaagcgcacaccgcgctgaaaatcaagagcacccgtaactacaactttgcgaagcaacgtctggaacagttcaaagagattcagagcctgaacaacctg<br>ctggttgtgaagaagctgaacgactttttcgatagcgaatttttcagcggcgaggaaacctacaccatctgcgttcaccatctgggtggcaaggacctgagcaaactgt<br>ataaggcgtgggaggatgatccggcggacccggaaaacgcgattgtggttctgtgcgacgatctgaaaaacaactttaagaaagagccgatccgtaacattctgcgt<br>tacatcttcaccattcgtcaagaatgcagcgcgcaggacatcctggcggcggcgaagtacaaccaacagctggatcgttataaaagccaaaaggcgaacccgag<br>cgttctgggtaaccagggctttacctggaccaacgcggtgatcctgccggagaaggcgcagcgtaacgaccgtccgaacagcctggatctgcgtatttggctgtacct<br>gaaactgcgtcacccggacggtcgttggaagaaacaccatatcccgttctacgatacccgtttcttccaagaaatttatgcggcgggcaacagcccggttgacacctg<br>ccagtttcgtaccccgcgtttcggttatcacctgccgaaactgaccgatcagaccgcgatccgtgttaacaagaaacatgtgaaagcggcgaagaccgaggcgcgta<br>ttcgtctggcgatccaacagggcaccctgccggtgagcaacctgaagatcaccgaaattagcgcgaccatcaacagcaaaggtcaagtgcgtattccggttaagttt<br>gacgtgggtcgtcaaaaaggcaccctgcagatcggtgaccgtttctgcggctacgatcaaaaccagaccgcgagccacgcgtatagcctgtgggaagtggttaaag<br>agggtcaataccataaagagctgggctgctttgttcgtttcatcagcagcggtgacatcgtgagcattaccgagaaccgtggcaaccaatttgatcagctgagctatga<br>aggtctggcgtacccgcaatatgcggactggcgtaagaaagcgagcaagttcgtgagcctgtggcagatcaccaagaaaaacaagaaaaaggaaatcgtgacc<br>gttgaagcgaaagagaagtttgacgcgatctgcaagtaccagccgcgtctgtataaattcaacaaggagtacgcgtatctgctgcgtgatattgttcgtggcaaaagcc<br>tggtggaactgcaacagattcgtcaagagatctttcgtttcattgaacaggactgcggtgttacccgtctgggcagcctgagcctgagcaccctggaaaccgtgaaagc<br>ggttaagggtatcatttacagctattttagcaccgcgctgaacgcgagcaagaacaacccgatcagcgacgaacagcgtaaagagtttgatccggaactgttcgcgct<br>gctggaaaagctggagctgattcgtacccgtaaaaagaaacaaaaagtggaacgtatcgcgaacagcctgattcagacctgcctggagaacaacatcaagttcatt<br>cgtggtgaaggcgacctgagcaccaccaacaacgcgaccaagaaaaaggcgaacagccgtagcatggattggttggcgcgtggtgtttttaacaaaatccgtcaa<br>ctggcgccgatgcacaacattaccctgttcggttgcggcagcctgtacaccagccaccaggacccgctggtgcatcgtaacccggataaagcgatgaagtgccgttg<br>ggcggcgatcccggttaaggacattggcgattgggtgctgcgtaagctgagccaaaacctgcgtgcgaaaaacatcggcaccggcgagtactatcaccaaggtgtt<br>aaagagttcctgagccattatgaactgcaggacctggaggaagagctgctgaagtggcgtagcgatcgtaaaagcaacattccgtgctgggtgctgcagaaccgtct<br>ggcggagaagctgggcaacaaagaagcggtggtttacatcccggttcgtggtggccgtatttattttgcgacccacaaggtggcgaccggtgcggtgagcatcgttttc<br>gaccaaaaacaagtgtgggtttgcaacgcggatcatgttgcggcggcgaacatcgcgctgaccgtgaagggtattggcgaacaaagcagcgacgaagagaacc<br>cggatggtagccgtatcaaactgcagctgaccagctaataaaagcttggtccattaggtagggacaatgattcagaaagtagaaattgcggcccaacgcaccggttt<br>gccattggaacgtgactcgaaggcaaaatgttcgtgatgtaatcgctttacaaacataaccttctgtggtaaaatattactcagttgcaaaatccgtctttcattgacggtgg<br>cccggtactacccccgactgagaaattcaacaaccctgctacaatgcagactgatctttgacaatttagttgcaatgcctaagaaatccgtctttcattgacgggtcatgg<br>tcctccttctttcattgacggcagtggcggaatagcggccctaagaaatccgtctttcattgacggcattcggcgctcgaataggaaatccgtctttcattgacggatcattc<br>atggtcctccttgtgttgcaacacctaagaaatccgtctttcattgacgggagccagccaaaaggcgtcgttgttggctctgccgccgttgcaacacctaagaaatccgtc<br>tttcattgacggggggaaactggcaacggcggacttccagcaacacctaagaaatccgtctttcattgacgggctgcccctcttggccgttgcaacacctaagaaatcc<br>gtctttcattgacgggtggcctcgacgggaacaacaggatcgtgggaggcccagcaacacctaagaaatccgtctttcattgacggttcacgccagcccagcaacac<br>ctaagaaatccgtctttcattgacgggctgcccctcttggccgttgcaacacctaagaaatccgtctttcattgacgggtcgagggattggaactggcgagaaatccgtct<br>ttcattgacgggtcgtaggcgacgaaaagcgttgcaaaacccaagaaatccgtctttcattggcatacagcctcgagagagaccatggccaccggtctctatcgaag<br>atatgaggagctcaaactcgagctgctaacaaagcccgaaaggaagctgagttggctgctgccaccgctgagcaataactagcataaccccttggggcctctaaac<br>gggtcttgaggggttttttgctgaaaggaggaactatatccgg

T7_Cas12i2_guide_nontarget	gaaattaatacgactcactataggttgcaaaacccaagaaatccgtctttcattgacgggtgattgcgcctgagcgagacgaaatacgcggttgcaaaacccaagaaatccgtctttcattgacggctaacccctctctaaacggaggggttt
T7_Cas12i2_guide_target1	gaaattaatacgactcactataggttgcaaaacccaagaaatccgtctttcattgacggtaaaggttgttttacgacagacgataacagggttgcaaaacccaagaaatccgtctttcattgacggctaacccctctctaaacggaggggttt
T7_Cas12i2_guide_target2	gaaattaatacgactcactataggttgcaaaacccaagaaatccgtctttcattgacggagtgtttcgccaagtacccgagccacatcaagttgcaaaacccaagaaatccgtctttcattgacggctaacccctctctaaacggaggggttt
T7_Cas12i2_guide_target3	gaaattaatacgactcactataggttgcaaaacccaagaaatccgtctttcattgacgggccaagtacccgagccacatcaaggatttctgttgcaaaacccaagaaatccgtctttcattgacggctaacccctctctaaacggaggggttt
T7_Cas12i2_guide_target4	gaaattaatacgactcactataggttgcaaaacccaagaaatccgtctttcattgacggtttaagagcgccatgccggaaggttatacccgttgcaaaacccaagaaatccgtctttcattgacggctaacccctctctaaacggaggggttt
T7_Cas12i2_guide_target5	gaaattaatacgactcactataggttgcaaaacccaagaaatccgtctttcattgacgggaaggcgacggcgtgtacaagacgcgtgctagttgcaaaacccaagaaatccgtctttcattgacggctaacccctctctaaacggaggggttt
T7_Cas12i2_guide_target6	gaaattaatacgactcactataggttgcaaaacccaagaaatccgtctttcattgacggtatctacaatcgtgtcacgctgactggtgaggttgcaaaacccaagaaatccgtctttcattgacggctaacccctctctaaacggaggggttt
T7_Cas12i2_guide_target7	gaaattaatacgactcactataggttgcaaaacccaagaaatccgtctttcattgacggtgcgtaagaacgttgcattccaatgcccgccgttgcaaaacccaagaaatccgtctttcattgacggctaacccctctctaaacggaggggttt

T7_Cas12i2_guide_target8	gaaattaatacgactcactataggttgcaaaacccaagaaatccgtctttcattgacggcaatgcccgccaagcattctgtatattctgcgttgcaaaacccaagaaatccgtctttcattgacggctaacccctctctaaacggaggggttt
T7_Cas12i2_guide_target9	gaaattaatacgactcactataggttgcaaaacccaagaaatccgtctttcattgacggtgcctgacaccgttaacaatggcatccgcgtgttgcaaaacccaagaaatccgtctttcattgacggctaacccctctctaaacggaggggttt
T7_Cas12i2_guide_target10	gaaattaatacgactcactataggttgcaaaacccaagaaatccgtctttcattgacggaaccaggcgtacgatattgaaggtgtgaccggttgcaaaacccaagaaatccgtctttcattgacggctaacccctctctaaacggaggggttt
T7_Cas12i2_guide_target11	gaaattaatacgactcactataggttgcaaaacccaagaaatccgtctttcattgacggaaccctgttatcgtctgtcgtaaaacaacctgttgcaaaacccaagaaatccgtctttcattgacggctaacccctctctaaacggaggggttt
T7_Cas12i2_guide_target12	gaaattaatacgactcactataggttgcaaaacccaagaaatccgtctttcattgacggtcaaacagttttgcaccttccgtcaatgccggttgcaaaacccaagaaatccgtctttcattgacggctaacccctctctaaacggaggggttt
T7_Cas12i2_guide_target13	gaaattaatacgactcactataggttgcaaaacccaagaaatccgtctttcattgacggataccttcgacgtcgccttccagttcggtgagttgcaaaacccaagaaatccgtctttcattgacggctaacccctctctaaacggaggggttt
T7_Cas12i2_guide_target14	gaaattaatacgactcactataggttgcaaaacccaagaaatccgtctttcattgacgggctttaatggtacccgtggtcgcgtcaccgggttgcaaaacccaagaaatccgtctttcattgacggctaacccctctctaaacggaggggttt
T7_Cas12i2_guide_target15	gaaattaatacgactcactataggttgcaaaacccaagaaatccgtctttcattgacgggaagctgatggtacgctcttgggtataacctgttgcaaaacccaagaaatccgtctttcattgacggctaacccctctctaaacggaggggttt
T7_Cas12i2_	gaaattaatacgactcactataggttgcaaaacccaagaaatccgtctttcattgacgggtaggtaaccatagcacgcgtcttgtacacggttgcaaaacccaagaaatccgtctttcattgacggctaacccctctctaaacggaggggttt

guide_target16	
T7_Cas12i2_guide_target17	gaaattaatacgactcactataggttgcaaaacccaagaaatccgtctttcattgacggtcaccagtcagcgtgacacgattgtagataggttgcaaaacccaagaaatccgtctttcattgacggctaacccctctctaaacggaggggttt
T7_Cas12i2_guide_target18	gaaattaatacgactcactataggttgcaaaacccaagaaatccgtctttcattgacggttaaagttctcaccagtcagcgtgacacgatgttgcaaaacccaagaaatccgtctttcattgacggctaacccctctctaaacggaggggttt
T7_Cas12i2_guide_target19	gaaattaatacgactcactataggttgcaaaacccaagaaatccgtctttcattgacggttacgcagaatgtgaccgtctttcttaaagtgttgcaaaacccaagaaatccgtctttcattgacggctaacccctctctaaacggaggggttt
T7_Cas12i2_guide_target20	gaaattaatacgactcactataggttgcaaaacccaagaaatccgtctttcattgacggggtcacaccttcaatatcgtacgcctggttggttgcaaaacccaagaaatccgtctttcattgacggctaacccctctctaaacggaggggttt
T7_SpCas9_Effector_wt	cccgcgaaattaatacgactcactataggggaattgtgagcggataacaattcccctctagaaataattttgtttaactttaagaaggagatttaaatatgaaaatcgaagaaggtaaaggtcaccatcaccatcaccacggatccatggataagaaatactcaataggcttagatatcggcacaaatagcgtcggatgggcggtgatcactgatgattataaggttccgtctaaaaagttcaaggttctgggaaatacagaccgccacagtatcaaaaaaaatcttataggggctcttttatttgacagtggagagacagcggaagcgactcgtctcaaacggacagctcgtagaaggtatacacgtcggaagaatcgtatttgttatctacaggagattttttcaaatgagatggcgaaagtagatgatagtttcttttcatcgacttgaagagtcttttttggtggaagaagacaagaagcatgaacgtcatcctatttttggaaatatagtagatgaagttgcttatcatgagaaatatccaactatctatcatctgcgaaaaaaattggcagattctactgataaagcggatttgcgcttaatctatttggccttagcgcatatgattaagtttcgtggtcatttttttgattgagggagatttaaatcctgataatagtgatgtggacaaactatttatccagttggtacaaacctacaatcaattatttgaagaaaaccctattaacgcaagtagagtagatgctaaagcgattctttctgcacgattgagtaaatcaagacgattagaaaatctcattgctcagctccccggtgagaagaaaaatggcttatttgggaatctcattgctttgttattgggattgacccctaattttaaatcaaattttgatttggcagaagatgctaaattacagctttcaaaagatacttacgatgatgatttagataatttattggcgcaaattggagatcaatatgctgatttgtttttggcagctaagaatttatcagatgctattttactttcagatatcctaagagtaaatagtgaaataactaaggctcccctatcagcttcaatgattaaacgctacgatgaacatcatcaagacttgactcttttaaaagctttagttcgacaacaacttccagaaaagtataaagaaatcttttttgatcaatcaaaaaacggatatgcaggttatattgatgggggagctagccaagaagaattttataaatttatcaaaccaattttagaaaaaatggatggtactgaggaattattggcgaaactaaatcgtgaagatttgctgcgcaagcaacggacctttgacaacggctctattccccatcaaattcacttgggtgagctgcatgctattttgagaagacaagaagacttttatccatttttaaaagacaatcgtgagaagattgaaaaaatcttgacttttcgaattccttattatgttggtccattggcgcgtggcaatagtcgttttgcatggatgactcggaagtctgaagaaacaattaccccatggaattttgaagaagttgtcgataaaggtgcttcagctcaatcatttattgaacgcatgacaaactttgataaaaatcttccaaatgaaaaagtactaccaaaacatagtttgctttatgagtattttacggtttataacgaattgacaaaggtcaaatatgttactgagggaatgcgaaaaccagcatttctttcaggtgaacagaagaaagccattgttgatttactcttcaaaacaaatcgaaaagtaaccgttaagcaattaaaagaagattatttcaaaaaaatagaatgttttgatagtgttgaaatttcaggcgttgaagatcggttta

	atgcgtcattaggtacctaccatgatttgctaaaaattatcaaagataaagatttttggataatgaagaaaatgaagatattttagaggatattgttttaacattgaccttatttgaagataaggaaatgattgaggaacgacttaaaaagtatgctcacctctttgatgataaggtgatgaaacagcttaaacgtcgccgttatactggttggggacgtttgtctcgaaaattgattaatggtattagggataagcaatctggcaaaacaatattagatttttgaaatcagatggttttgccaatcgcaattttatgcagctgatccatgatgatagtttgacatttaaagaagatattcaaaaagcacaagtgtctggacaaggcgatagtttacatgaacatattgcaaatttagctggtagccctgctattaaaaaaggtattttacagactgtaaaagttgttgatgaattggtcaaagtaatggggcggcataagccagaaaatatcgttattgaaatggcacgtgaaaatcagacaactcaaaagggccagaaaaattcgcgtgagcgtatgaaacgtattgaagaaggaataaaagaactaggaagtgatattctaaaggagtatcctgttgaaaacactcaattacaaaatgaaaagctctatctctattatctccaaaatggaagagacatgtatgtggaccaagaattagatattaatcgtttaagtgattatgatgtcgatcacattgttccacaaagtttccttaaagacgattcaatagacaataaggtgttaacgcgttctgataaaaatcgtggtaaatcggataacgttccaagtgaagaagtagtcaaaaagatgaaaaactattggaaacaacttctaaacgccaagttaatcactcaacgtaagtttgataatttaacaaaagctgaacgtggaggtttgagtgaacttgataaagctggttttatcaaacgccaattggttgaaactcgccaaatcactaagcatgtggcacaaattttggatagtcgcatgaatactaaatacgatgaaaatgataaacttattcgagaggttagagtgattacctaaaatctaaattagtttctgacttccgaaaagatttccaattctataaagtacgtgagattaacaattaccatcatgcccatgatgcgtatcttaatgccgtcgttggaactgctttgattaagaaatatccaaaacttgaatcggagtttgtctatggtgattataaagtttatgatgttcgtaaaatgattgctaagtctgagcaggaaataggcaaagcaaccgcaaaatatttcttttactctaatatcatgaacttcttcaaaacagaaattacacttgcaaatggagagattcgcaaacgccctctaatcgaaactaatggggaaactggagaaattgtctgggataaagggcgagattttgccacagtgcgcaaagtattgtccatgccccaagtcaatattgtcaagaaaacagaagtacagacaggcggattctccaaggagtcaattttaccaaaaagaaattcggacaagcttattgctcgtaaaaaagactgggatccaaaaaaatatggtggttttgatagtccaacggtagcttattcagtcctagtggttgctaaggtggaaaaagggaaatcgaagaagttaaaatccgttaaagagttactagggatcacaattatggaaagaagttcctttgaaaaaaatccgattgactttttagaagctaaaggatataaggaagttagaaaagacttaatcattaaactacctaaatatagtctttttgagttagaaaacggtcgtaaacggatgctggctagtgccggagaattacaaaaaggaaatgagctggctctgccaagcaaatatgtgaatttttatatttagctagtcattatgaaaagttgaagggtagtccagaagataacgaacaaaaacaattgtttgtggagcagcataagcattatttagatgagattattgagcaaatcagtgaattttctaagcgtgttattttagcagatgccaatttagataaagttcttagtgcatataacaaacatagagacaaaccaatacgtgaacaagcagaaaatattattcatttatttacgttgacgaatcttggagctcccgctgcttttaaatattttgatacaacaattgatcgtaaacgatatacgtctacaaaagaagttttagatgccactcttatccatcaatccatcactggtctttatgaaacacgcattgatttgagtcagctaggaggtgactaataaaaagcccgaaaggaagctgagttggctgctgccaccgctgagcaataactagcataaccccttggggcctctaaacgggtcttgaggggttttttgctgaaaggaggaactatatccgg
T7_SpCas9_guide_nontarget	gaaattaatacgactcactataggggtgattgcgcctgagcgagagttttagagctagaaatagcaagttaaaataaggctagtccgttatcaacttgaaaaagtggcaccgagtcggtgctttttaacccctctctaaacggaggggttt
T7_SpCas9_guide_target1	gaaattaatacgactcactataggggaactggaaggcgacgtcgagttttagagctagaaatagcaagttaaaataaggctagtccgttatcaacttgaaaaagtggcaccgagtcggtgctttttaacccctctctaaacggaggggttt
T7_SpCas9_g	gaaattaatacgactcactatagggcaagtacccgagccacatcagttttagagctagaaatagcaagttaaaataaggctagtccgttatcaacttgaaaaagtggcaccgagtcggtgctttttaacccctctctaaacggaggggttt

uide_target2	
T7_SpCas9_guide_target3	gaaattaatacgactcactatagggcgtgtacaagacgcgtgctagttttagagctagaaatagcaagttaaaataaggctagtccgttatcaacttgaaaaagtggcaccgagtcggtgctttttaacccctctctaaacggaggggttt
T7_SpCas9_guide_target4	gaaattaatacgactcactatagggctgcctgacaccgttaacaagttttagagctagaaatagcaagttaaaataaggctagtccgttatcaacttgaaaaagtggcaccgagtcggtgctttttaacccctctctaaacggaggggttt
T7_SpCas9_guide_target5	gaaattaatacgactcactatagggtgaaggtgtgaccgaaaaacgttttagagctagaaatagcaagttaaaataaggctagtccgttatcaacttgaaaaagtggcaccgagtcggtgctttttaacccctctctaaacggaggggttt
T7_SpCas9_guide_target6	gaaattaatacgactcactatagggaccttccgtcaatgccgtcagttttagagctagaaatagcaagttaaaataaggctagtccgttatcaacttgaaaaagtggcaccgagtcggtgctttttaacccctctctaaacggaggggttt
T7_SpCas9_guide_target7	gaaattaatacgactcactatagggtcttaaagaaatccttgatggttttagagctagaaatagcaagttaaaataaggctagtccgttatcaacttgaaaaagtggcaccgagtcggtgctttttaacccctctctaaacggaggggttt
T7_SpCas9_guide_target8	gaaattaatacgactcactataggggccgtcgccttcgaagctgagttttagagctagaaatagcaagttaaaataaggctagtccgttatcaacttgaaaaagtggcaccgagtcggtgctttttaacccctctctaaacggaggggttt
T7_SpCas9_guide_target9	gaaattaatacgactcactatagggggcagaatatacagaatgctgttttagagctagaaatagcaagttaaaataaggctagtccgttatcaacttgaaaaagtggcaccgagtcggtgctttttaacccctctctaaacggaggggttt
T7_SpCas9_guide_target10	gaaattaatacgactcactatagggcaccttcaatatcgtacgccgttttagagctagaaatagcaagttaaaataaggctagtccgttatcaacttgaaaaagtggcaccgagtcggtgctttttaacccctctctaaacggaggggttt

T7_forward_primer	cccgcgaaattaatacgactcactataggg
T7term_reverse_primer	caaaaaacccctcaagacccgtttagaggccccaaggggttatgctag

Supplementary Table 3. A list of DNA sequences used in transient transfection screens and validation in HEK293T cells

Name	Format	PAM	Spacer
Cas12i2 _AAVS1_T1	Plasmid	CTTT	TGTCCCCCCAAGTTTTGGAC
Cas12i2 _AAVS1_T2	Plasmid	CTTT	GTGAGAATGGTGCGTCCTAG
Cas12i2_AAVS1_T3	Plasmid	CTTT	AACTGGCCCTGGCTTTGGCA
Cas12i2_AAVS1_T4	Plasmid	CTTT	GTAGCCTCTCCCGCTCTGGT
Cas12i2_AAVS1_T5	Plasmid	CTTT	GGGAAGTGGTTGGTCAGCAT
Cas12i2_AAVS1_T6	Plasmid	CTTT	AGCGGGTATGGGAAGGGCTT
Cas12i2_EMX1_T1	Plasmid	CTTT	GGGCGCAGGGCCACCTGGAC
Cas12i2_EMX1_T2	Plasmid	CTTT	GGATGGCGACTTCAGGCACA
Cas12i2_EMX1_T3	Plasmid	CTTT	GGGGAGGCCTGGAGTCATGG
Cas12i2_EMX1_T4	Plasmid	CTTT	GAGCCAGTGTTGCTAGTCAA
Cas12i2_EMX1_T5	Plasmid	CTTT	AGCAAGGGACTATTCAGGGA
Cas12i2_EMX1_T6	Plasmid	CTTT	AAAATTGAGCAATCTACCCT
Cas12i2_VEGFA_T1	Plasmid	CTTT	TGGGGGTGACCGCCGGAGCG
Cas12i2_VEGFA_T2	Plasmid	CTTT	AATCCTCCACCAGTCATGGT
Cas12i2_VEGFA_T3	Plasmid	CTTT	GTTGACATTGTCCACACCTG
Cas12i2_VEGFA_T4	Plasmid	CTTT	TTAAACTCTCCATGGACCAG
Cas12i2_VEGFA_T5	Plasmid	CTTT	GCCCATACTGGGGACCAAGG
Cas12i2_VEGFA_T6	Plasmid	CTTT	GCCGTAACCCTTCGTGGGTA
SpCas9_AAVS1_T1	Plasmid	TGG	GCTTTTGTCCCCCCAAGTTT
SpCas9_AAVS1_T2	Plasmid	AGG	TTGTGAGAATGGTGCGTCCT
SpCas9_AAVS1_T3	Plasmid	TGG	GCTTTAACTGGCCCTGGCTT
SpCas9_AAVS1_T4	Plasmid	TGG	CTTTGTAGCCTCTCCCGCTC
SpCas9_AAVS1_T5	Plasmid	TGG	TGGGAAGTGGTTGGTCAGCA
SpCas9_AAVS1_T6	Plasmid	GGG	GACTTTAGCGGGTATGGGAA
SpCas9_EMX1_T1	Plasmid	TGG	TCTTTGGGCGCAGGGCCACC
SpCas9_EMX1_T2	Plasmid	AGG	TGGATGGCGACTTCAGGCAC
SpCas9_EMX1_T3	Plasmid	TGG	TTTGGGGAGGCCTGGAGTCA
SpCas9_EMX1_T4	Plasmid	GGG	GAGCCAGTGTTGCTAGTCAA
SpCas9_EMX1_T5	Plasmid	GGG	CTTTAGCAAGGGACTATTCA
SpCas9_EMX1_T6	Plasmid	TGG	TAAAATTGAGCAATCTACCC
SpCas9_VEGFA_T1	Plasmid	CGG	TGGGGGTGACCGCCGGAGCG
SpCas9_VEGFA_T2	Plasmid	TGG	CTTTAATCCTCCACCAGTCA
SpCas9_VEGFA_T3	Plasmid	TGG	TTGTTGACATTGTCCACACC
SpCas9_VEGFA_T4	Plasmid	AGG	TTTTAAACTCTCCATGGACC
SpCas9_VEGFA_T5	Plasmid	AGG	TTTGCCCATACTGGGGACCA
SpCas9_VEGFA_T6	Plasmid	GGG	GCTTTGCCGTAACCCTTCGT
Cas12i2_AAVS1_Screen_1	PCR Amplicon	TTTG	TGAGAATGGTGCGTCCTAGG
Cas12i2_AAVS1_Screen_2	PCR Amplicon	GTTG	AGCCCCTCCAGCCGGTCCTG
Cas12i2_AAVS1_Screen_3	PCR Amplicon	CTTG	TGGCCTGGGTCACCTCTACG
Cas12i2_AAVS1_Screen_4	PCR Amplicon	GTTG	GTGGAGTCCAGCACGGCGCG
Cas12i2_AAVS1_Screen_5	PCR Amplicon	GTTG	GGTGAGGGAGGAGAGATGCC
Cas12i2_AAVS1_Screen_6	PCR Amplicon	CTTA	TCTGGTGACACACCCCCATT
Cas12i2_EMX1_Screen_1	PCR Amplicon	CTTC	GTGAGTGGCTTCCCTGCCGC
Cas12i2_EMX1_Screen_2	PCR Amplicon	TTTG	TGGATAAAACTTCTCGGAGG
Cas12i2_EMX1_Screen_3	PCR Amplicon	CTTG	TGTGACCTGTTCCCACATCT
Cas12i2_EMX1_Screen_4	PCR Amplicon	ATTG	TGTGAGGGCCTAGTGGGGTG
Cas12i2_EMX1_Screen_5	PCR Amplicon	ATTG	AGAGGTGGGAATCAGGCCCA
Cas12i2_EMX1_Screen_6	PCR Amplicon	GTTC	TCTTGAGATGGGCTCGGGCT
Cas12i2_VEGFA_Screen_1	PCR Amplicon	GTTG	GTGCCCTTCGGTCCTCGGCA
Cas12i2_VEGFA_Screen_2	PCR Amplicon	ATTC	TGTGCCCGTGGGGACCCCCG
Cas12i2_VEGFA_Screen_3	PCR Amplicon	GTTG	GTGCCCGCTGCTGTCTAATG
Cas12i2_VEGFA_Screen_4	PCR Amplicon	TTTC	GGAGGCCCGACCGGGGCCGG
Cas12i2_VEGFA_Screen_5	PCR Amplicon	CTTG	TGAGTGCTCCGTGTTAAGGG
Cas12i2_VEGFA_Screen_6	PCR Amplicon	TTTC	TCCGCTCTGAGCAAGGCCCA
SpCas9_AAVS1_Screen_1	PCR Amplicon	AGG	TTGTGAGAATGGTGCGTCCT
SpCas9_AAVS1_Screen_2	PCR Amplicon	TGG	TGAGCCCCTCCAGCCGGTCC
SpCas9_AAVS1_Screen_3	PCR Amplicon	CGG	TGTGGCCTGGGTCACCTCTA
SpCas9_AAVS1_Screen_4	PCR Amplicon	CGG	TGGTGGAGTCCAGCACGGCG

SpCas9_AAVS1_Screen_5	PCR Amplicon	CGG	GGTGAGGGAGGAGAGATGCC
SpCas9_AAVS1_Screen_6	PCR Amplicon	TGG	GGTGACACACCCCCATTTCC
SpCas9_EMX1_Screen_1	PCR Amplicon	CGG	CGTGAGTGGCTTCCCTGCCG
SpCas9_EMX1_Screen_2	PCR Amplicon	AGG	TTGTGGATAAAACTTCTCGG
SpCas9_EMX1_Screen_3	PCR Amplicon	TGG	GTGTGACCTGTTCCCACATC
SpCas9_EMX1_Screen_4	PCR Amplicon	TGG	GAGATTGTGTGAGGGCCTAG
SpCas9_EMX1_Screen_5	PCR Amplicon	AGG	CGTATTGAGAGGTGGGAATC
SpCas9_EMX1_Screen_6	PCR Amplicon	CGG	GTGTTCTCTTGAGATGGGCT
SpCas9_VEGFA_Screen_1	PCR Amplicon	CGG	GGTTGGTGCCCTTCGGTCCT
SpCas9_VEGFA_Screen_2	PCR Amplicon	CGG	TCTGTGCCCGTGGGGACCCC
SpCas9_VEGFA_Screen_3	PCR Amplicon	TGG	CCCGCTGCTGTCTAATGCCC
SpCas9_VEGFA_Screen_4	PCR Amplicon	CGG	TTCGGAGGCCCGACCGGGGC
SpCas9_VEGFA_Screen_5	PCR Amplicon	AGG	CTTGTGAGTGCTCCGTGTTA
SpCas9_VEGFA_Screen_6	PCR Amplicon	AGG	TGCTTTCTCCGCTCTGAGCA

124

Supplementary Table 4. A list of DNA sequences used in *in vitro* cleavage and EMSA

Name	target	gRNA
Cas12i2 Target	TCCATGTCTCGTTATACGCTGTGGTTCGCCAACGCACG<br>AGTGTACTACTAACGCTGCGTCAGCTTTGGCGTCAACA<br>TGGGTGACTATCACCTGAATCATCAGCAACTACTCGAG<br>TGTTTGACAGCTAGCTCAGTCCTAGGTATAAT	GTTGCAAAACCCAAGAAATCCGTCTTTC<br>ATTGACGGGGAGCGCAGTCACCAAAAC<br>TTGTCCTTTCAGGTTGCAAAACCCAAGA<br>AATCCGTCTTTCATTGACGGAGGTGATA<br>GTCACCCATGTTGACGCCAAAGCGTTG<br>CAAAACCCAAGAAATCCGTCTTTCATTG<br>ACGG
Cas12i2 Non-Target	TCCATGTCTCGTTATACGCTGTGGTTCGCCAACGCAGA<br>ACCACACTACTAACTGCCAAAGAGTGCCGTTTTGGCTA<br>TCGCGACAGTATTTTTAAACATGAATACACTACTGAACC<br>ACTTGACAGCTAGCTCAGTCCTAGGTATAAT	
cis ssDNA target (Cas12i2)	GTGGTTCGCCAACATATAGTTCACTACTAGCATGCTGC<br>CCTTGACTAGCAACACTGGCTCAAAGAAGGAAAGACTA<br>CTTATAGTTCTTGACAGCTAGCTC	AGAAATCCGTCTTTCATTGACGGGAGC<br>CAGTGTTGCTAGTCAA
trans ssDNA target (Cas12i2)	GCCTGGTCCATGGAGAGTTTAAAAAGTCTTTTGGACTA<br>CTGGAACGACTTGACAGCTAGC	
AAVS target 2 Cut site target	TCCATGTCTCGTTATACGCTGTGGTTCGCCAACAACAC<br>TAGTACTACTGATAAATTACCCCCCAAGTCCCTCACCTC<br>TCCAAAGCTGCCCATACTACTACACTAGTTTGACAGCTA<br>GCTCAGTCCTAGGTATAATGCTAGC	AGAAATCCGTCTTTCATTGACGGGGAG<br>AGGTGAGGGACTTGGG (Cas12i2)<br>GGAGAGGTGAGGGACTTGGGGTTTTAG<br>AGCTAGAAATAGCAAGTTAAAATAAGGC<br>TAGTCCGTTATCAACTTGAAAAAGTGGC<br>ACCGAGTCGGTGC (SpCas9)
EMX target 6 Cut site target	TCCATGTCTCGTTATACGCTGTGGTTCGCCAACATATAG<br>TTCACTACTAGCATGCTGCCCTTGACTAGCAACACTGG<br>CTCAAAGAAGGAAAGACTACTTATAGTTCTTGACAGCTA<br>GCTCAGTCCTAGGTATAATGCTAGC	AGAAATCCGTCTTTCATTGACGGGAGC<br>CAGTGTTGCTAGTCAA (Cas12i2)
VEGFA target 5 Cut site target	TCCATGTCTCGTTATACGCTGTGGTTCGCCAACGCAGG<br>AACGACACTACTAGCTGGATGAGCCTGGTCCATGGAGA	AGAAATCCGTCTTTCATTGACGGTTAAA<br>CTCTCCATGGACCAG (Cas12i2)

	GTTTAAAAAGTCTTTTGGACTACTGGAACGACTTGACAG<br>CTAGCTCAGTCCTAGGTATAATGCTAGC	
AAVS1 target 5 EMSA target	TTTCTGTCTGCAGCTTGTGGCCTGGGTCACCTCTACGG<br>CTGGCCCAGATCCTTCCCTGCCGCCTCCTTCAGGTTCC<br>GTCTTCCTCCACTCCCTCTTCCCCTTGCTCTCTGCTGTG<br>TTGCTGCCCAAGGATGCTCTTTCCGGAGCACTTCCTTC<br>TCGGCGCTGCACCACGTGATGTCCTCTGAGCGGATCC<br>TCCCCGTGTCTGGGTCCTCTCCGGGCATCTCTCCTCCC<br>TCACCCAACCCCATGCCGTCTTCACTCGCTGGGTTCCC<br>TTTTCCTTCTCCTTCTGGGGCCTGTGCCATCTCTCGTTT<br>CTTAGGATGGCCTTCTCCGACGGATGTCTCCCTTGCGT<br>CCCGCCTCCCCTTCTTGTAGGCCTGCATCATCACCGTT<br>TTTCTGGACAACCCCAAAGTACCCCGTCTCCCTGGCTT<br>TAGCCACCTCTCCATCCTCTTGCTTTCTTTGCCTGGACA<br>CCCCGTTCTCCTGTGGATTCGGGTCACCTCTCACTCCT<br>TTCATTTGGGCAGCTCCCCTACCCCCCTTACCTCTCTA<br>GTCTGTGCTAGCTCTTCCAGCCCCCTGTCATGGCATCT<br>TCCAGGGGTCCGAGAGCTCAGCTAGTCTTCTTCCTCCA<br>ACCCGGGCCCCTATGTCCACTTCAGGACAGCATGTTTG<br>C	AGAAATCCGTCTTTCATTGACGGGGGA<br>AGTGGTTGGTCAGCAT
VEGFA target 2 EMSA target	GACCTTAATACTTACCATGGCTTTGGACCAGGGAACTA<br>GGGGGATAGTGAGAGCAGGGAGAGGGAAGTGTGGGG<br>AAGGTACAGGGGACCTCGACAGTGAAGCATTCTGGGG<br>TTTTCCTCCTGCATTTCGAGCTCCCCAGCCCCCAACAT<br>CTGGTTAGTCTTTAACTTCCTCGGGTTCATAACCATAGC<br>AGTCCAGGAGTGGTGGGCATATTCTGTGCCCGTGGGG<br>ACCCCCGGTTGTGTCCTGTTCGACTCAGAAGACTTGGA<br>GAAGCCAGAGGCTGTTGGTGGGAGGGAAGTGAGGAG<br>GGAGGAGGGGCTGGGTGGCTGGGCCTGTGCACCCCA<br>GCCCCTGCCCATGCCCATGCCTTGCTCTCTTTCTGTCC<br>TCAGTGGTCCCAGGCTGCACCCATGGCAGAAGGAGGA<br>GGGCAGAATCATCACGAAGGTGAGTCCCCCTGGCTGT<br>TGGATGGGGTTCCCTGTCCTCTCAGGGGATGGGTGGA<br>TGGCCTAATTCCTTTTTCTTCAGAACTGTGGGGAGGAA<br>G	AGAAATCCGTCTTTCATTGACGGAATCC<br>TCCACCAGTCATGGT

Supplementary Table 5. A list of RNA sequences used in RNP ex vivo editing

Name	target	gRNA
ABR-001 B2M 1 crRNA	B2M	rArGrArArArUrCrCrGrUrCrUrUrUrCrArUrUrGrArCrGrGrArArUrGrUrCrGrGrArUrGrGrArUrGrArArArCrC
ABR-001 B2M 2 crRNA	B2M	rArGrArArArUrCrCrGrUrCrUrUrUrCrArUrUrGrArCrGrGrCrUrArUrCrUrCrUrUrGrUrArCrUrArCrArCrUrG
ABR-001 B2M 3 crRNA	B2M	rArGrArArArUrCrCrGrUrCrUrUrUrCrArUrUrGrArCrGrGrArCrArCrGrGrCrArGrGrCrArUrArCrUrCrArUrC
ABR-001 B2M 4 crRNA	B2M	rArGrArArArUrCrCrGrUrCrUrUrUrCrArUrUrGrArCrGrGrGrUrCrArCrArGrCrCrCrArArGrArUrArGrUrUrA
ABR-001 TRAC crRNA	TRAC	rArGrArArArUrCrCrGrUrCrUrUrUrCrArUrUrGrArCrGrGrArGrArGrUrCrUrCrUrCrArGrCrUrGrGrUrArCrA
ABR-001 CIITA crRNA	CIITA	rArGrArArArUrCrCrGrUrCrUrUrUrCrArUrUrGrArCrGrGrUrUrArArCrArGrCrGrArUrGrCrUrGrArCrCrCrC
ABR-001 BCL11A 1 crRNA	BCL11A enhancer	rArGrArArArUrCrCrGrUrCrUrUrUrCrArUrUrGrArCrGrGrGrArArGrCrUrArGrUrCrUrArGrUrGrCrArArGrC
ABR-001 BCL11A 2 crRNA	BCL11A enhancer	rArGrArArArUrCrCrGrUrCrUrUrUrCrArUrUrGrArCrGrGrCrUrGrGrArGrCrCrUrGrUrGrArUrArArArArGrC
ABR-001 BCL11A 3 crRNA	BCL11A enhancer	rArGrArArArUrCrCrGrUrCrUrUrUrCrArUrUrGrArCrGrGrUrArCrCrCrCrArCrCrCrArCrGrCrCrCrCrCrArC
SpCas9 BCL11A sgRNA	BCL11A enhancer	mC*mU*mA*rArCrArGrUrUrGrCrUrUrUrUrArUrCrArCrGrUrUrUrUrArGrArGrCrUrArGrArArArUrArGrCrArArGrUrUrArArArArUrArArGrGrCrUrArGrUrCrCrGrUrUrArUrCrArArCrUrUrGrArArArArArGrUrGrGrCrArCrCrGrArGrUrCrGmG*mU*mG*rC

Supplementary Table 6. A list of antibodies used in flow cytometry

Antibody	Clone	Fluorophore	Protein	Vendor	Catalog #	Dilution
Anti-human B2M	2M2	PE	Human B2M	BioLegend	316305	1:20
Anti-human TRAC	IP26	APC/Cy7	Human TRAC	BioLegend	306728	1:20
Anti-human HLA-DR/DP/DQ	Tü39	FITC	Human HLA-DR/DP/DQ	BioLegend	361706	1:20
Anti-human CD45	HI30	APC	Human CD45	BioLegend	304012	1:20
Anti-mouse CD45	30-F11	BV785	Mouse CD45	BioLegend	103149	1:40
Anti-human CD15	HI98	PE	Human CD15	BioLegend	301906	1:20
Anti-human CD66b	G10F5	PE	Human CD66b	BioLegend	305106	1:20
Anti-human CD19	HIB19	BV421	Human CD19	BioLegend	302234	1:20
Anti-human CD20	2H7	BV421	Human CD20	BioLegend	302330	1:20
Anti-human CD71	CY1G4	FITC	Human CD71	BioLegend	334104	1:20
Anti-human HbF	HBF-1	APC	Human HbF	Thermofisher	MHFH05	1:20
Anti-human HbF	HBF-1	FITC	Human HbF	Thermofisher	MHFH04	1:20

Engineered Cas12i2 is a versatile high-efficiency platform for therapeutic genome editing

McGaw, C., et al.

Supplementary Figures

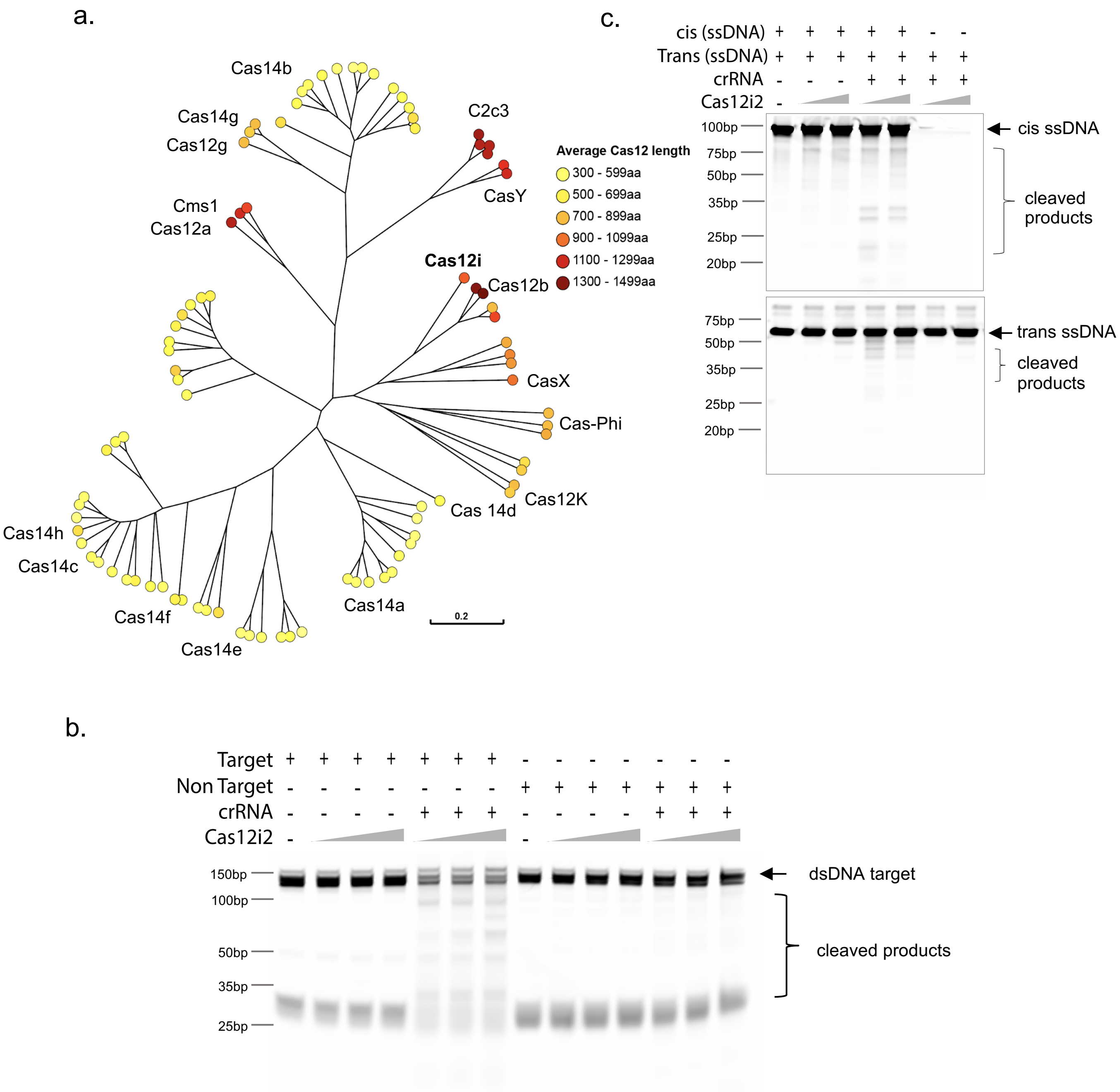

Supplementary Fig. 1. Type V-I nucleases Cas12i2 exhibited *in vitro* target-dependent dsDNA and ssDNA cleavage. a. Phylogenetic tree of diverse CRISPR Cas type V systems with Cas12i (bold, subtype V-I) representing an evolutionarily distinct branch. Average Cas12 effector lengths are colored. b. *In vitro* crRNA dependent cleavage of IR-labelled target (with PAM and complementary spacer) and non-target (with PAM and non-complementary spacer) dsDNA by Cas12i2. c. *In vitro* cleavage of cis target (ssDNA target with complementarity to crRNA, top panel) and trans ssDNA (with no complementarity to crRNA, bottom panel). Cis and trans ssDNA are orthogonally fluorescent labelled and visualized separately in respective fluorescence channels. shown are representative images of denaturing urea gels from n=2 experiments. Source Data are provided as a Source Data file.

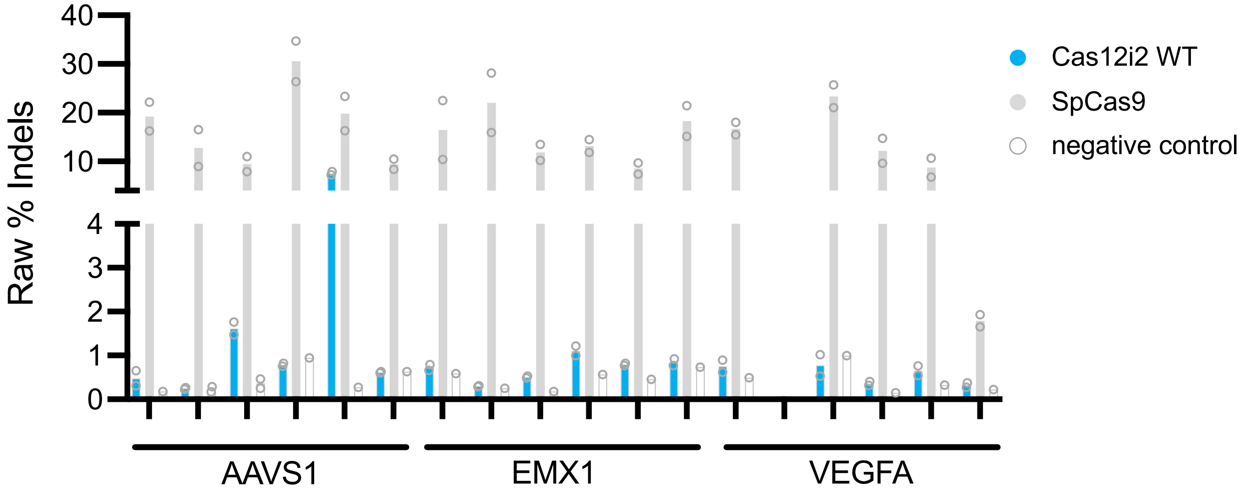

Supplementary Fig. 2. Indel activity of Cas12i2 and SpCas9 at individual 18 target sites in HEK293T cells. Each column represents one target site, each dot represent one replicate. Source Data are provided as a Source Data file.

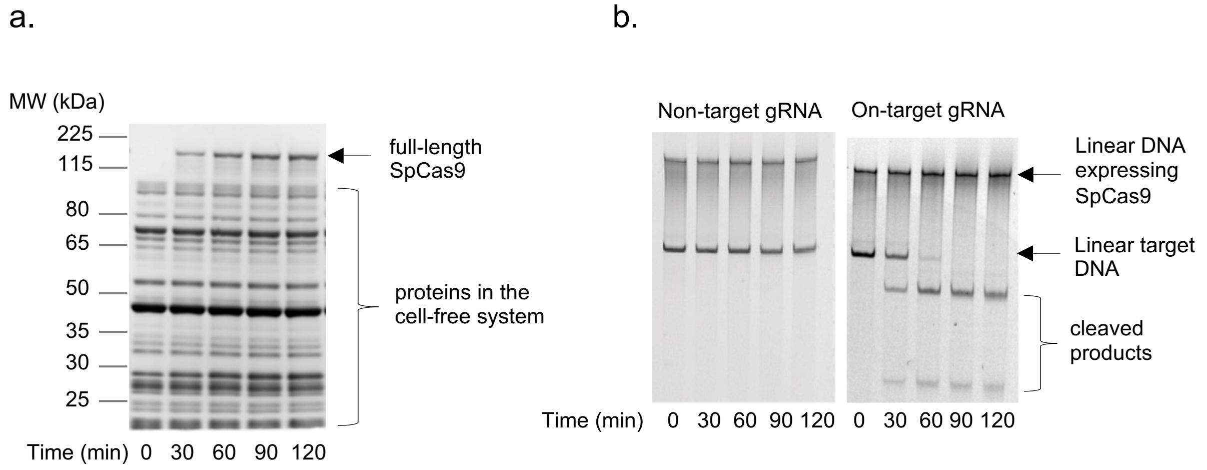

Supplementary Fig. 3. Cell-free expression and *in vitro* cleavage activity of SpCas9. a. Coomassie stained SDS-PAGE gel showing time course of *in vitro* expression of SpCas9 in the reconstituted cell-free protein synthesis system. Gel is from n=1 experiments. . b. *In vitro* cleavage of non-target (left panel) and target (right panel) linear dsDNA by cell-free expressed SpCas9 (from aliquots of reactions in (a)). DNA fragments are resolved on 6% TBE gel. Some carry-over linear DNA expressing SpCas9 and possibly genomic DNA fragments from the cell-free system are visible on the upper part of gel. Shown are representative images of SDS-PAGE gels from n=2 experiments. Source Data are provided as a Source Data file.

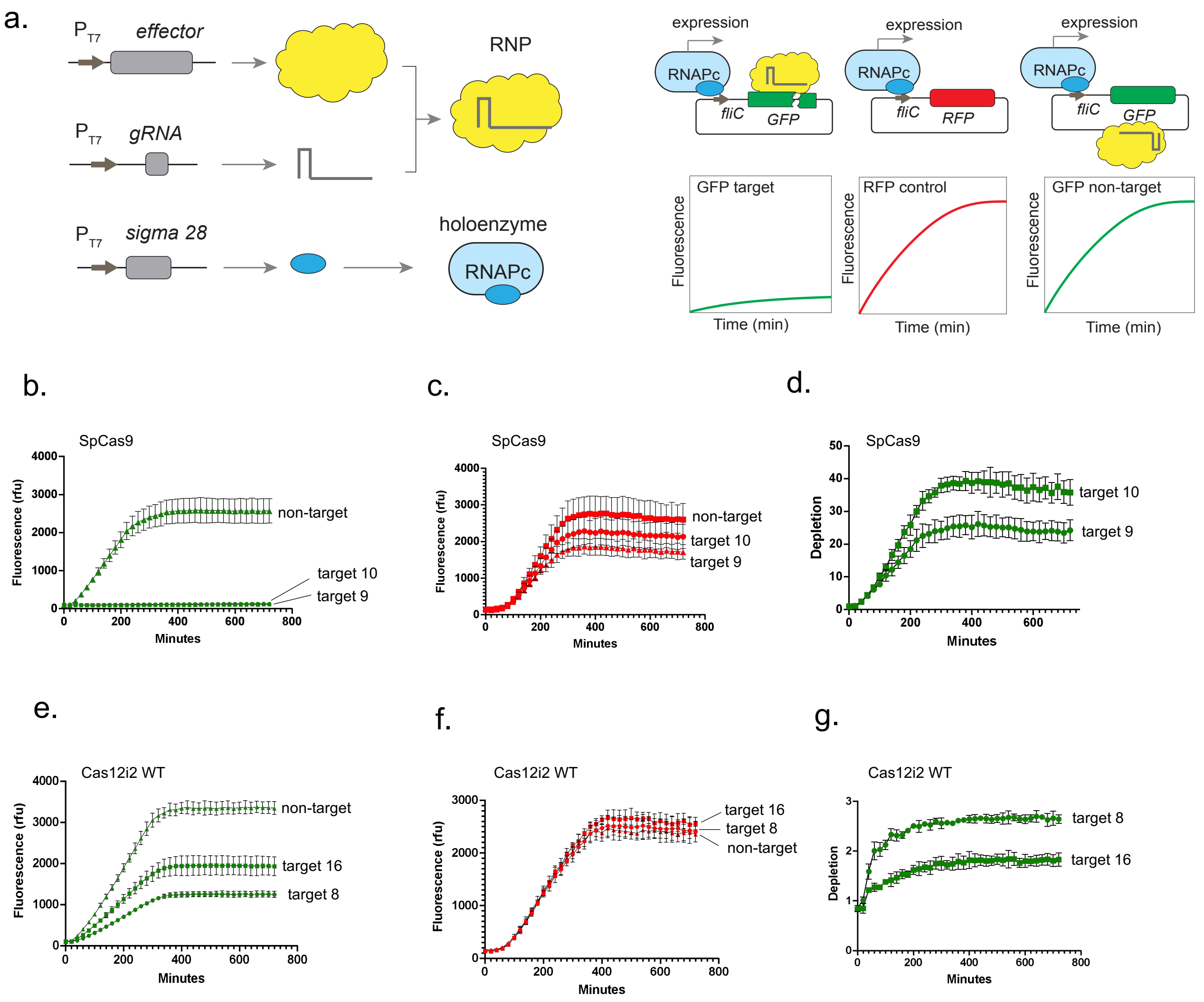

Supplementary Fig. 4. *In vitro* fluorescent reporter assay for rapid cell-free measurement of *in vitro* activity of Cas12i2 and SpCas9. a. Schematic description of the assay. A CRISPR Cas effector and gRNAs are expressed from T7 promoter (P_{T7}) by T7 RNP polymerase to form a ribonucleoprotein complex (RNP). GFP and RFP reporters are expressed from a sigma 28-specific promoter (fliC) by E. coli RNA polymerase holoenzyme consisting of RNA polymerase core enzyme (RNAPc) and sigma 28 transcription factor, expressed from T7 promoter. When GFP target gRNA is expressed (from T7 promoter), RNP cleaves the GFP coding region before and/or during GFP expression, resulting in poor GFP expression and low GFP fluorescence. When GFP non-target gRNA is expressed, RNP cleaves a site outside of the GFP gene without affecting GFP expression. The RFP expression is not affected by RNP interference; therefore, RFP serves as an expression and fluorescence control for normalizing well-to-well variations. b-c. Time course measurement of GFP (b) and RFP (c) fluorescence of *in vitro* reactions expressing SpCas9 and gRNAs for two GFP target sites (targets 9 and 10) and one non-target site. Shapes represent mean and error bars represent s.d. of n=3 replicates (target 9, 10) or n=12 replicates (non-target). d. GFP signal decrease (depletion) of SpCas9 for the two GFP target sites plotted as ratios of non-target over target GFP fluorescence normalized by RFP fluorescence. Shapes represent mean and error bars represent s.d. of n=3 replicates. e-f. Time course measurement of GFP (e) and RFP (f) fluorescence of *in vitro* reactions expressing Cas12i2 WT and pre-crRNAs for two GFP target sites (targets 8 and 16) and one non-target site. Shapes represent mean and error bars represent s.d. of n=3 replicates (target 8, 16) or n=12 replicates (non-target). g. GFP signal decrease (depletion) of Cas12i2 WT for the two GFP target sites plotted as ratios of non-target over target GFP fluorescence normalized by RFP fluorescence. Shapes represent mean and error bars represent s.d. of n=3 replicates. Source Data are provided as a Source Data file.

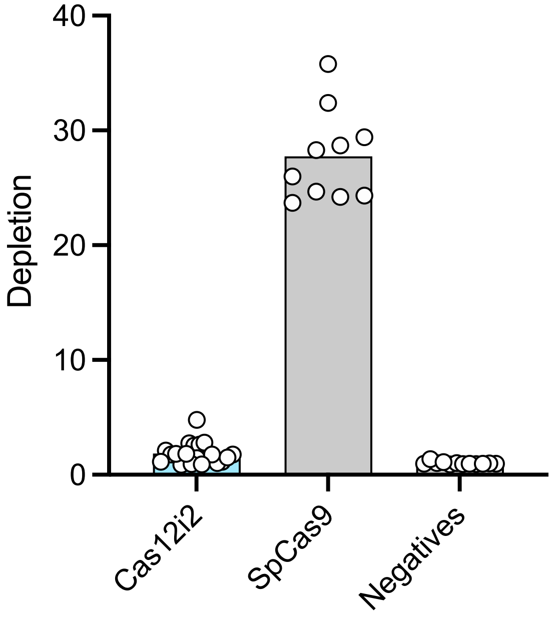

Supplementary Fig. 5. Validation of SpCas9 and Cas12i2 WT in *in vitro* fluorescent reporter assay with additional targets. *In vitro* activities of wild type Cas12i2 and SpCas9 at 20 and 10 GFP target sites, respectively, were measured by GFP signal decrease (depletion) in the assay. Each dot represents a single target site, averaged from n=3 replicates (n=1 for negatives) with s.d. <10%. Each bar represents the mean across all GFP targets. Source Data are provided as a Source Data file.

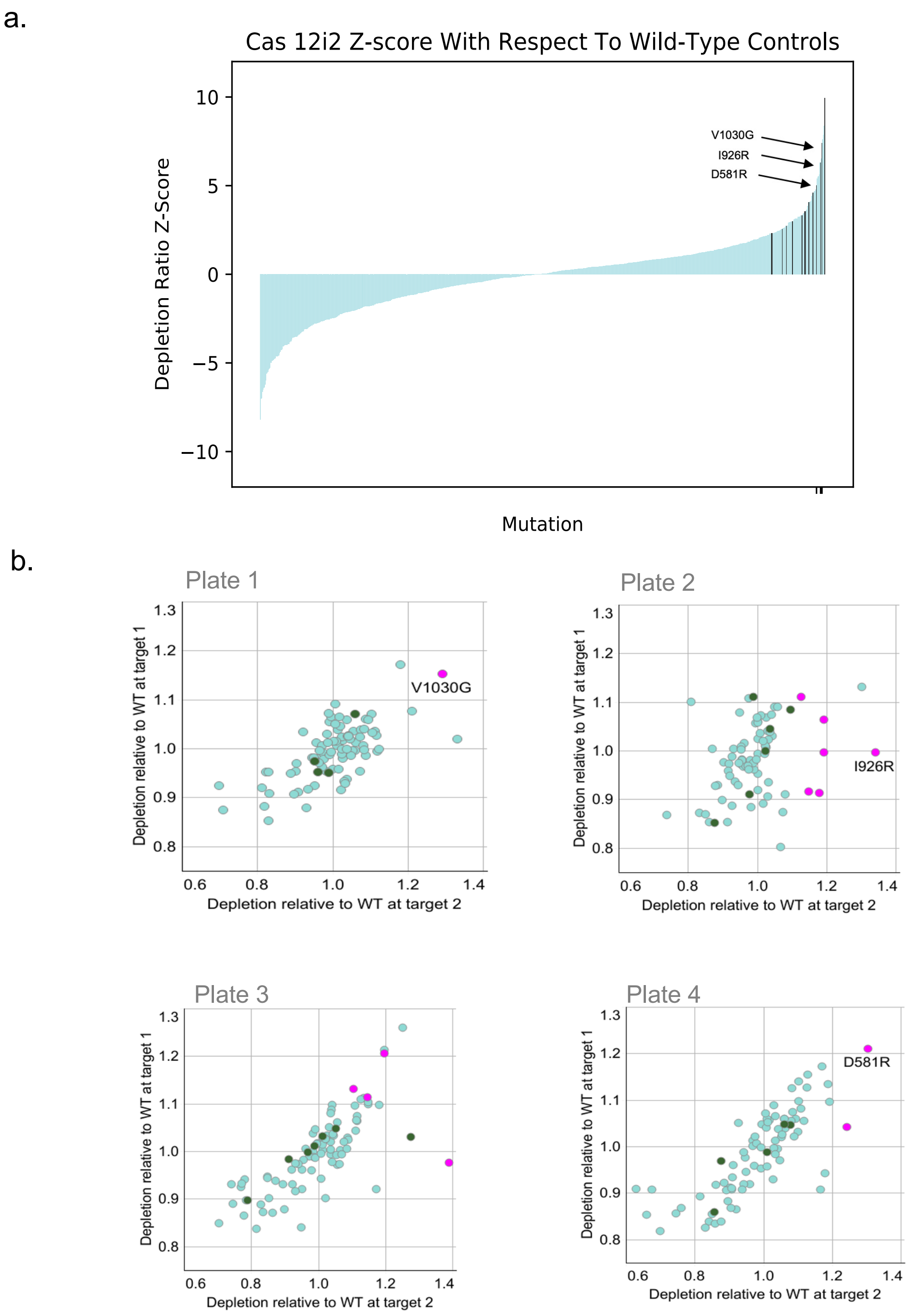

Supplementary Fig. 6. *In vitro* screen of arginine and glycine substitutions in the fluorescent reporter assay. a. Z-score of GFP signal decrease (depletion) relative to the means of WT controls for all arginine and glycine variants screened in the FDA, listed in ascending order. Black bars indicate the the 14 variants tested in HEK293T. b. GFP signal decrease (depletion) relative to the mean of WT controls for arginine and glycine single-substitution variants at two GFP target sites (target 1 and 2) measured from four separate 384-well plates (plates 1-4). The wild type controls (arginine-to-arginine and glycine-to-glycine substitutions) on each plate are colored in green. The single-substitution variants chosen for indel measurement in mammalian cells are colored in magenta. Each circle represents a single variant, averaged from n=3 replicates with s.d. less than 20%. Three substitutions in ABR-001 are indicated. Source Data are provided as a Source Data file.

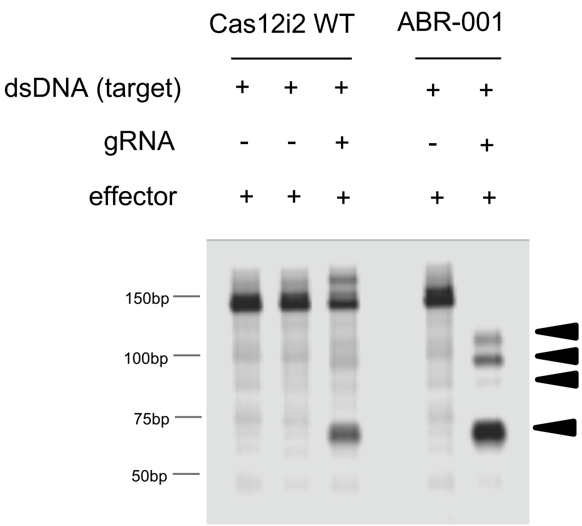

Supplementary Fig. 7. *In vitro* cleavage of target dsDNA by purified Cas12i2 WT and ABR-001. Black arrows represent cleavage products. The gel is a representative image of n=2 experiments. Cas12i2 WT and ABR-001 were at final concentrations of 1μM and 0.5μM, respectively. Source Data are provided as a Source Data file.

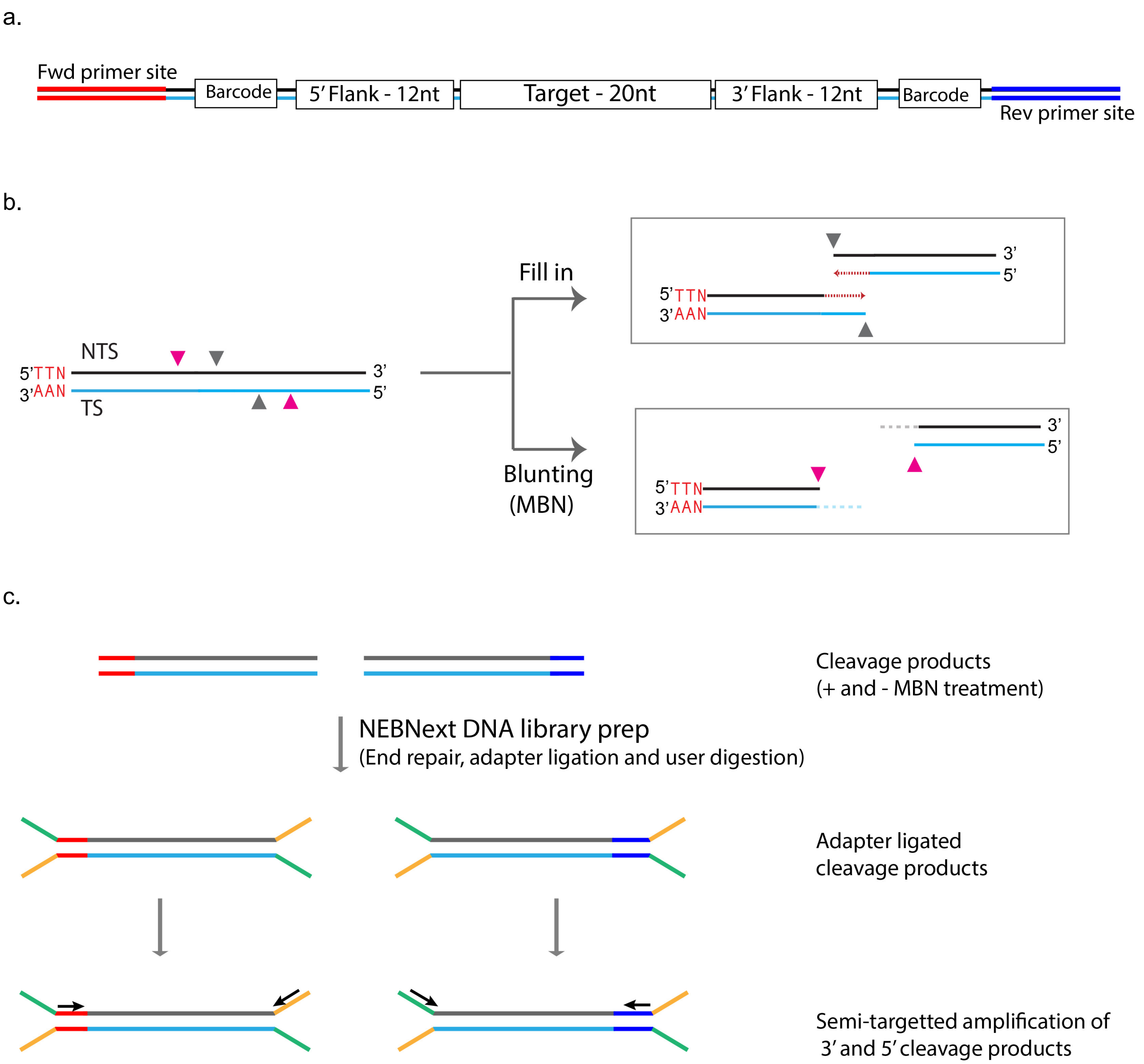

Supplementary Fig. 8. Cut site analysis of Cas12i2 *in vitro* cleavage products. a. Design of oligos containing target sequences for cut site analysis. In addition to target sequence and 12 nt of flank sequence on both ends of the target, the oligos contain internal barcodes and priming sites to allow for targeted amplification. b. All cleavage products are split into two halves where one half is treated with mung bean nuclease (MBN), which blunts the 5' and 3' overhangs (blunting treatment). The other half reaction is end repaired (part of NEBNext DNA library prep) which fills the 5' overhangs (fill in treatment) and blunts any 3' overhangs. Both halves are then progressed to library prep and semi-targeted amplification. Cut sites with 5' overhangs as indicated by grey arrows are captured by the fill in treatment which results in fill in of any 5' overhangs. Therefore the 5' and 3' sequencing of these products will indicate cut sites on template and non-template strand respectively (TS, NTS). Additional cut sites shown in pink may be captured by a blunting method that blunts all 5' and 3' overhangs. As a result, 5' cleavage products in blunting method indicate any additional cut sites on the NTS and the 3' cleavage products indicate additional cut sites on the TS c. Semi-targeted amplification after NEBNext adapter ligation allows for specific amplification of 5' and 3' cleavage products. The amplified products are pooled and read out by NGS.

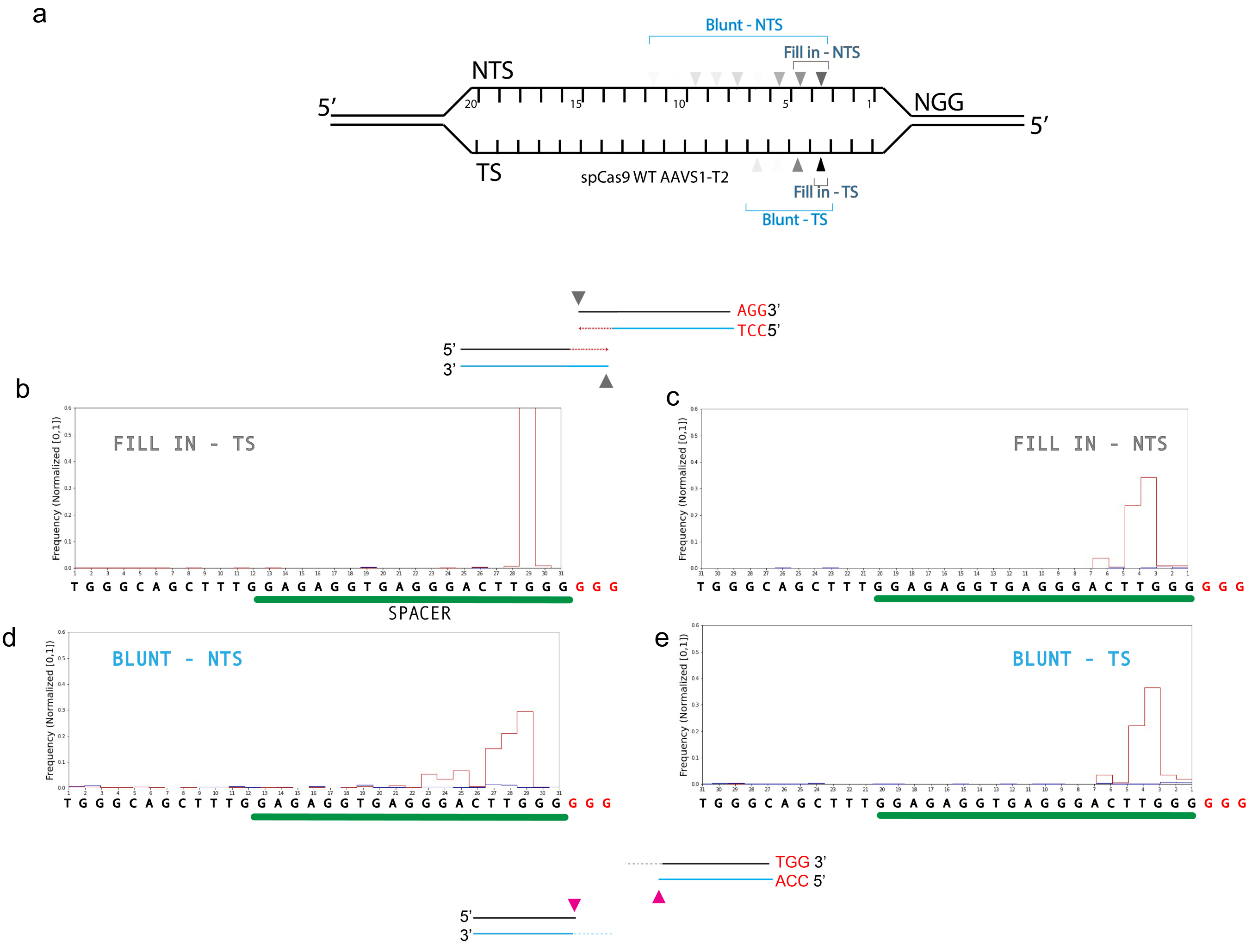

Supplementary Fig. 9. Cut site analysis of Cas9 cleavage products for AAVS1-T2. NGS read out of Cas9 cleavage products. b-e. Histogram of read lengths obtained from semi-targeted amplification of 5’ and 3’ cleavage products. b and d show read length histograms of 5’ cleavage products for fill-in and blunting treatment respectively. c and e show read length histograms for 3’ cleavage cleavage products for fill-in and blunting treatment respectively. Each read length histogram is mapped to the target sequence as shown on the x-axis. The PAM is indicted in red. The cut sites obtained form read length histograms are illustrated in the R-loop diagram shown in (a). 5’ and 3’ read length histograms for fill-in treatment indicate the cut site on template strand (TS) and Non-template strand (NTS) respectively. 5’ and 3’ read length histograms for blunting treatment indicate the cut site on non-template strand (NTS) and template strand (TS) respectively

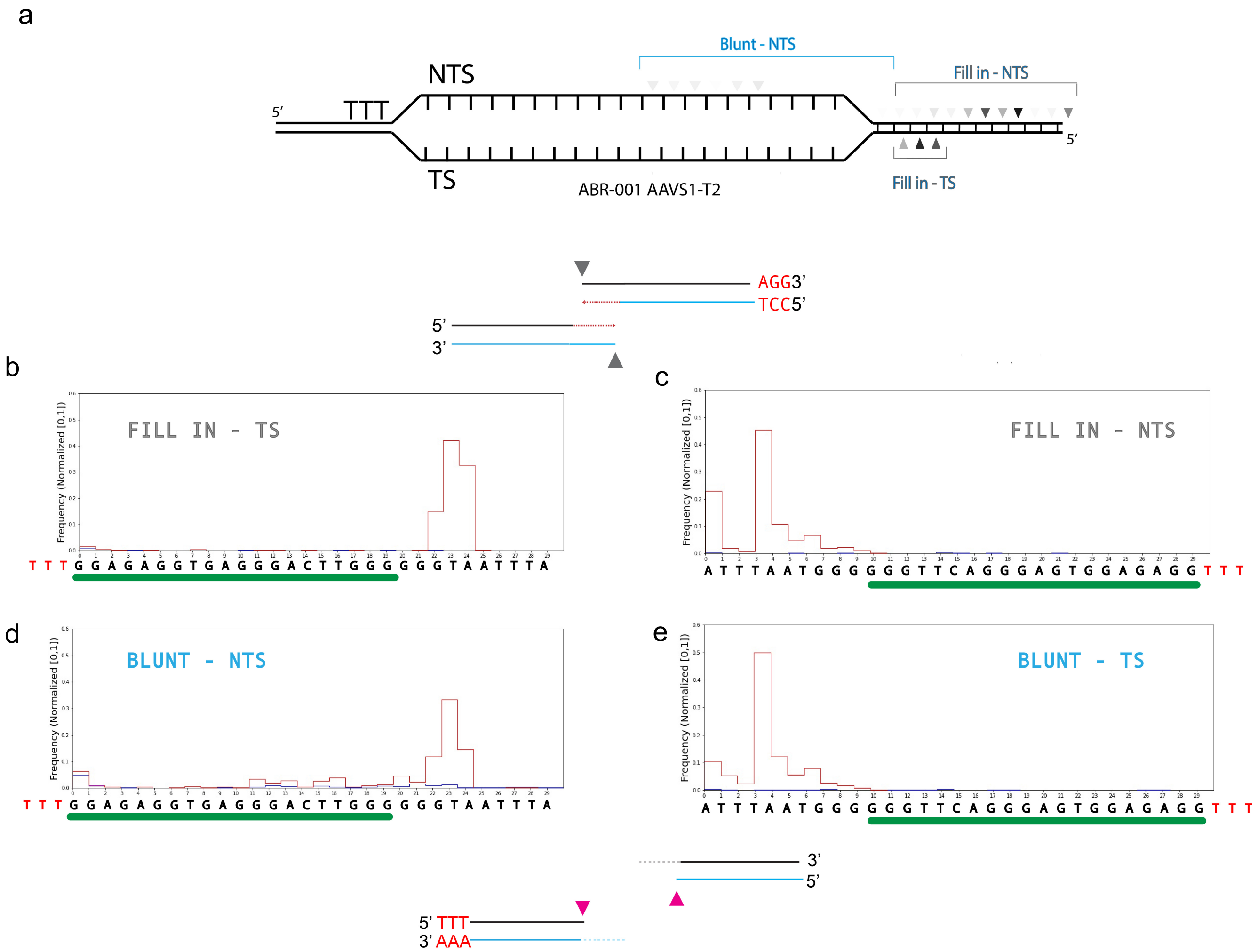

Supplementary Fig. 10. Cut site analysis of ABR-001 cleavage products for AAVS1-T2. NGS readout of ABR-001 cleavage products. b-e. Histogram of read lengths obtained from semi-targeted amplification of 5' and 3' cleavage products. b and d show read length histograms of 5' cleavage products for fill-in and blunting treatment, respectively. c and e show read length histograms for 3' cleavage cleavage products for fill-in and blunting treatment, respectively. Each read length histogram is mapped to the target sequence as shown on the x-axis. The PAM is indicted in red. The cut sites obtained form read length histograms are illustrated in the R-loop diagram shown in (a). 5' and 3' read length histograms for fill-in treatment indicate the cut site on template strand (TS) and Non-template strand (NTS) respectively. 5' and 3' read length histograms for blunting treatment indicate the cut site on non-template strand (NTS) and template strand (TS) respectively. Since blunting treatment blunts both 5' and 3' overhangs, the cut sites resulting from fig e (3' blunted cleavage products) could result either from the cuts on NTS (already captured by the fill in method or on the TS. Due to this ambiguity the cut sites indicated in fig. e were not assigned to either strand.

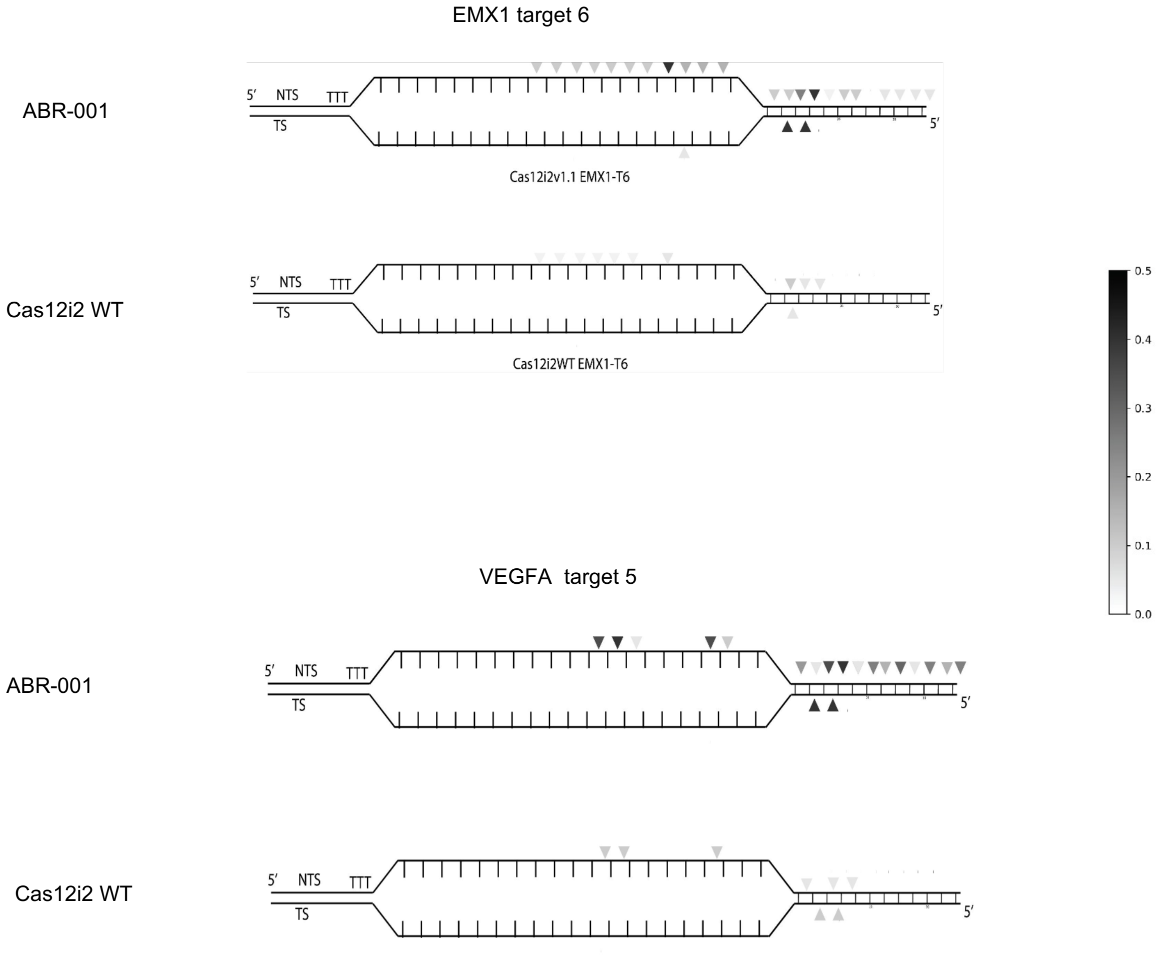

Supplementary Fig. 11. Additional *in vitro* cut site analysis for ABR-001 and Cas12i2 WT. Shown are *in vitro* cleavage sites (triangles) of ABR-001 and Cas12i2 WT (TTT PAM) on the template (TS) and non-template (NTS) strands of EMX1 target 6 and VEGFA target 5, analyzed by deep sequencing. The scale bar (right) represents the cleavage frequency as measured by the number of sequencing reads.

Supplementary Fig. 12

a.

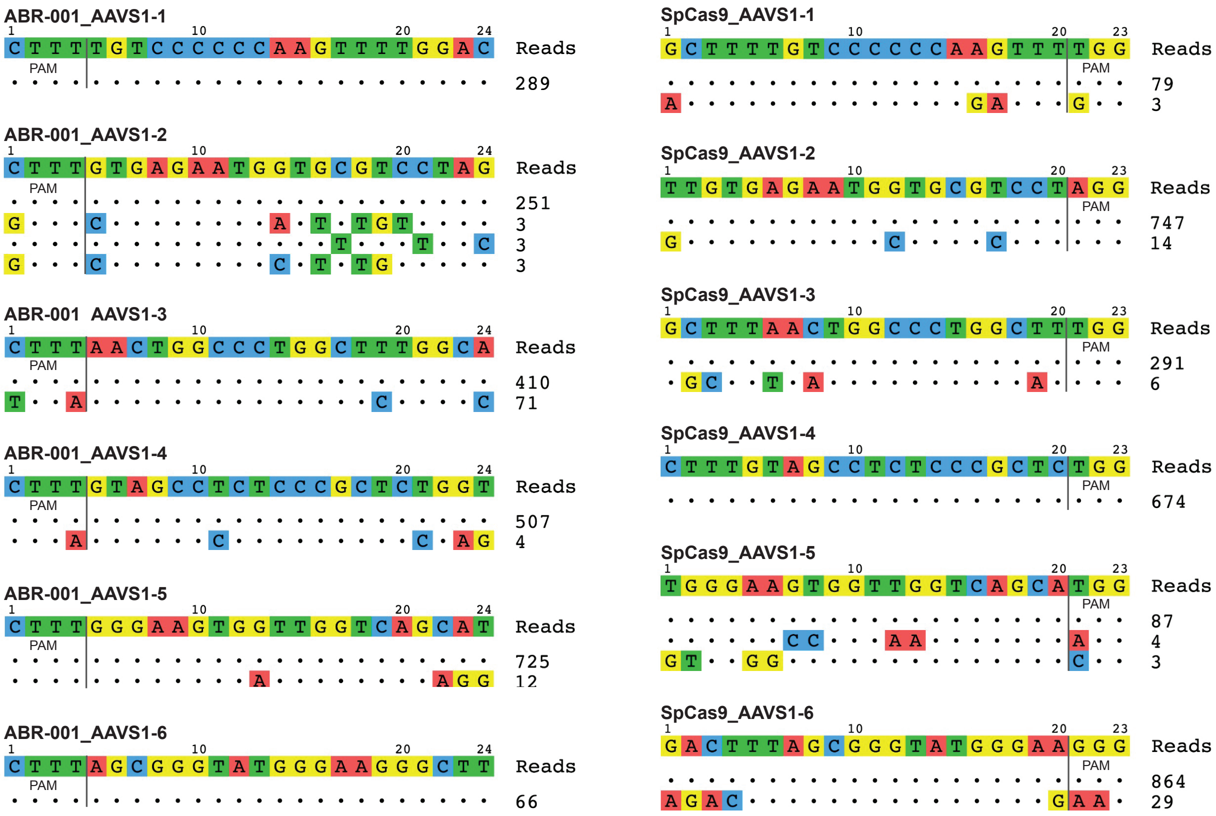

Supplementary Fig. 12

b.

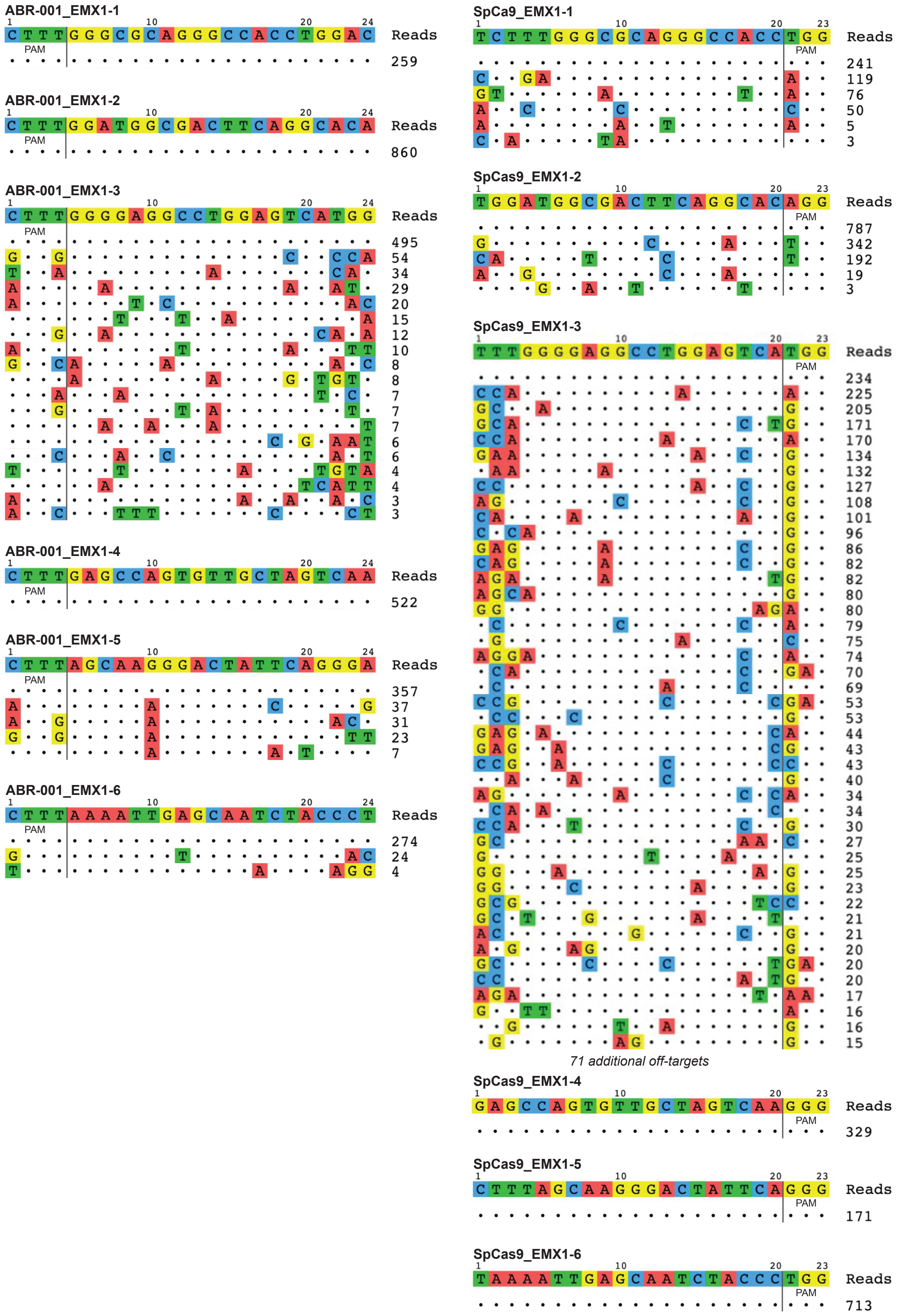

Supplementary Fig. 12

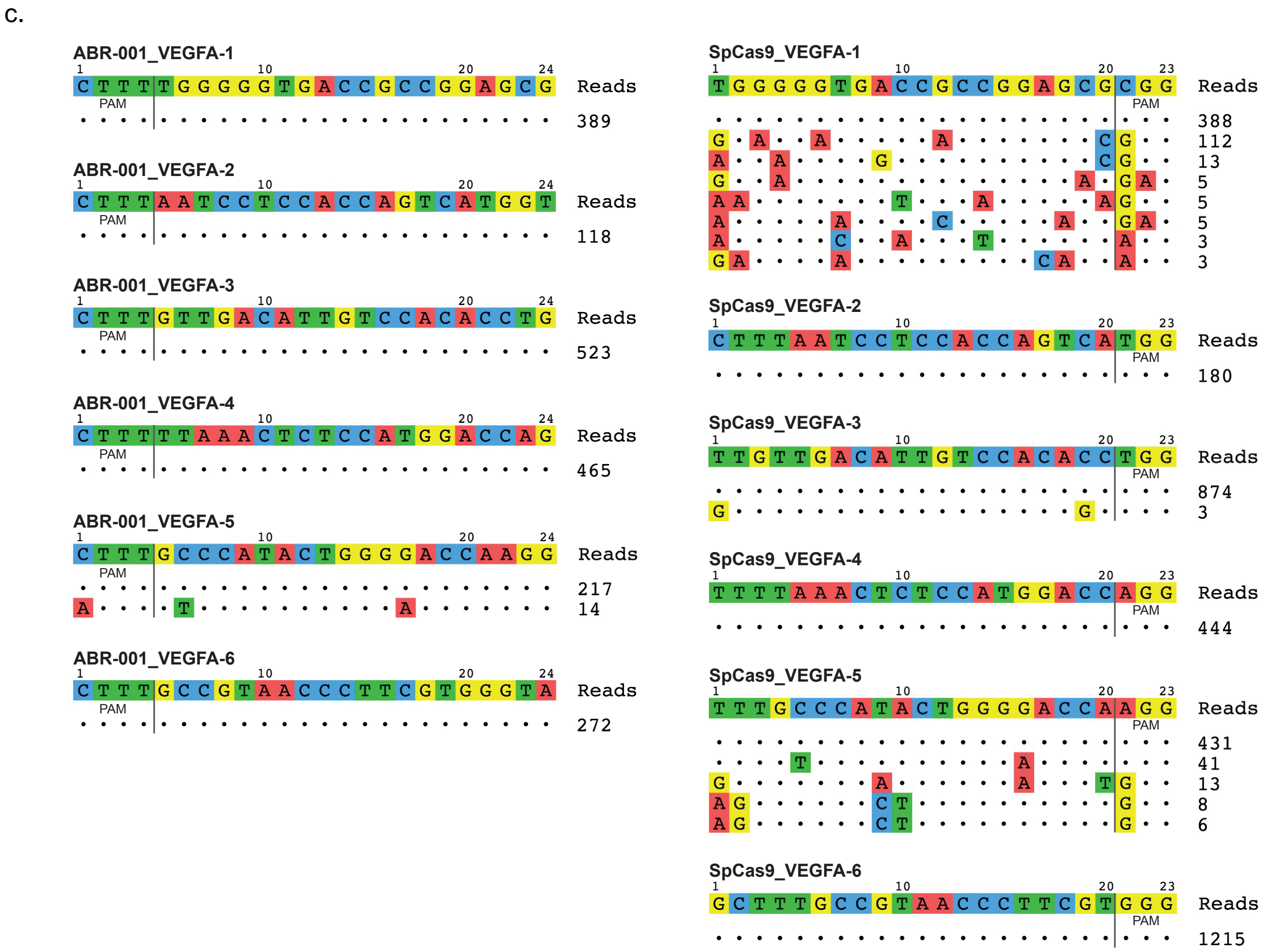

Supplementary Fig. 12. Additional TTISS data of ABR-001 and SpCas9 at six targets for each of AAVS1 (a), EMX1 (b), and VEGFA (c). Dots indicate a nucleotide at which the off-target matches the on target. Letters with a colored background indicate the identify of a mismatch. Read counts represents unique reads.

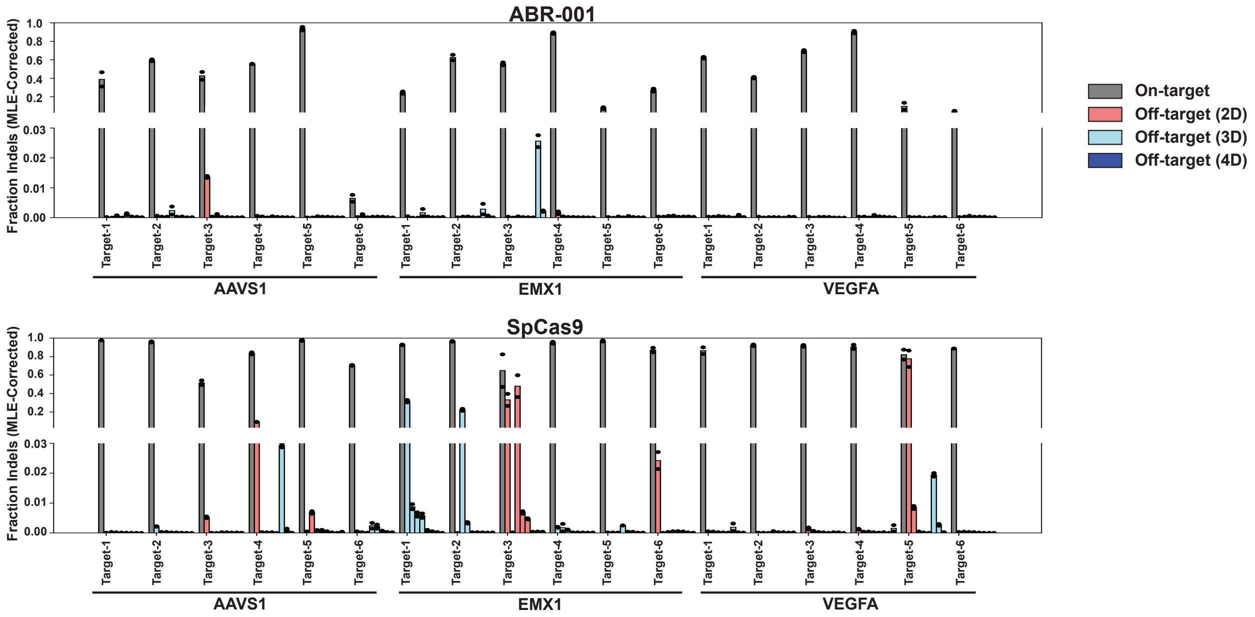

Supplementary Fig. 13. Measurement of computationally-predicted off targets for ABR-001 and SpCas9 delivered by RNP. Gray bars indicate fraction of reads showing indels at on-targets. Colored bars indicate fraction of reads showing indels at off targets of levenshtein distance 2D (pink), 3D (light blue), and 4D (dark blue). Technical background has been removed by maximum likelihood estimation. Source Data are provided as a Source Data file.

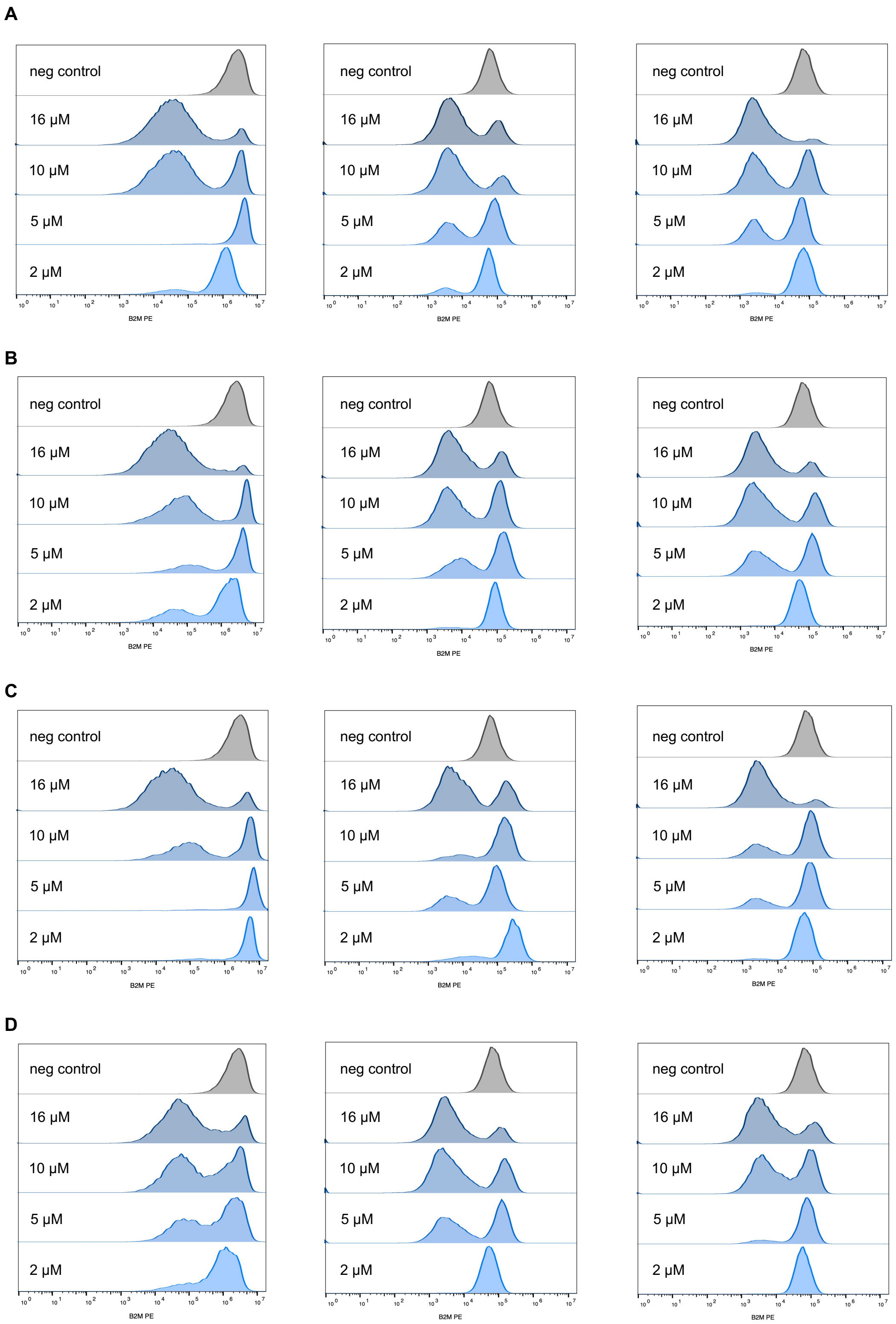

Supplementary Fig. 14. ABR-001 enables protein knockdown of B2M in T cells. Histograms show B2M protein level knockdown at 7 days post electroporation with ABR-001 RNP with B2M guide 1 (A), guide 2 (B), guide 3 (C), or guide 4 (D) at decreasing concentrations (16µM, 10µM, 5µM, and 2µM). Each panel represents one technical replicate. Y-axes represents count normalized to mode.

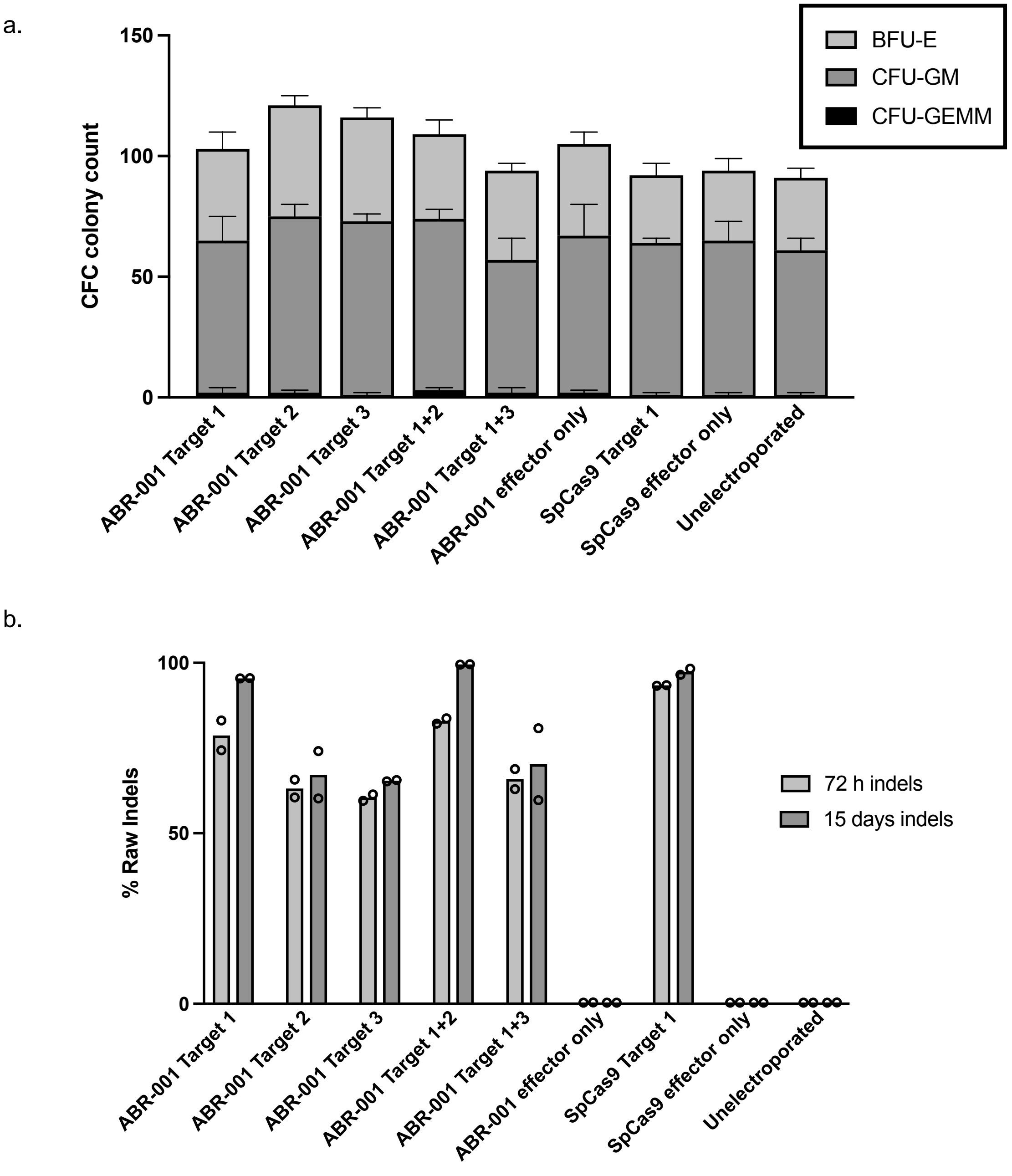

Supplementary Fig. 15. ABR-001 edited CD34+ HSPCs maintain indels and multilineage reconstitution. a. CFC colony counts at day 15 post plating. Erythroid (BFU-E), myeloid (CFU-GM), and mixed (CFU-GEMM) colony counts are shown. Bars represent the average colony counts of three technical replicates from a representative donor, and error bars represent the standard deviation of the mean for three technical replicates b. Indel rates at the BCL11A enhancer in CD34+ HSPCs at 72 h post electroporation and in isolated CFC colonies at 15 days post plating. ABR-001 RNP and effector only samples were transfected at a final concentration of 16 µM, and SpCas9 RNP and effector only samples were transfected at a final concentration of 5 µM. Bars at 72 h represent the average of two biological replicates (i.e. two separate donors). Bars at 15 days represent the average indels from 24 CFU-GM colonies 12 BFU-E sequenced colonies. Each dot represents a single replicate. Source Data are provided as a Source Data file.

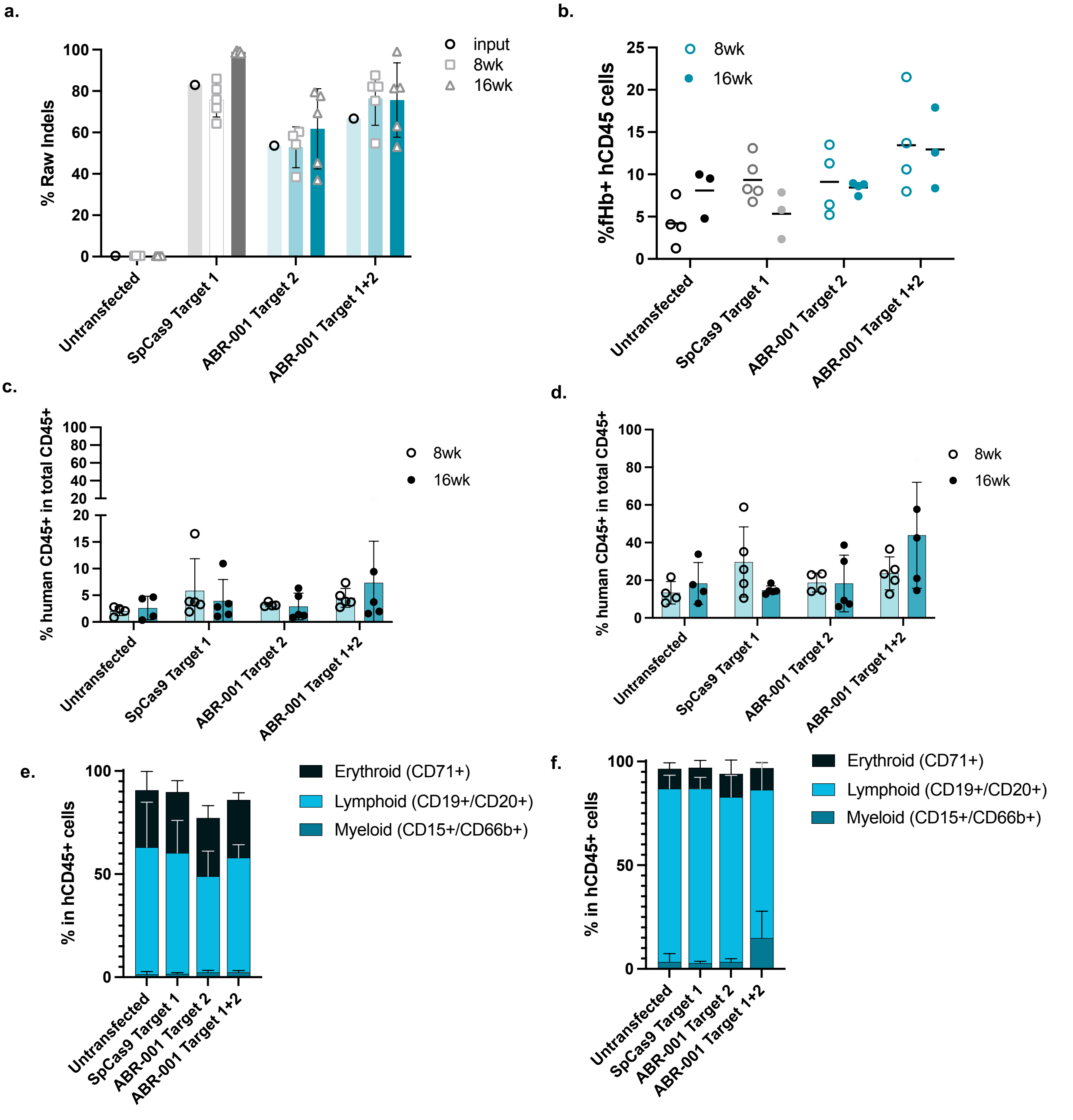

Supplementary Fig. 16. ABR-001 edited cells retain engraftment capacity and functionality, upon transplantation in immunodeficient mice. a. Indel rates at the time of adoptive transfer and 8 and 16 weeks post-transplantation of hCD34+ cells, edited with guides targeting the BCL11a enhancer and adoptively transferred into sub-lethally irradiated NOD-SCID mice at a dose of 200,000 cells/mouse. Bars for 8 and 16 weeks represent the mean and error bars represent s.d. of n=4 (Untransfected 8wk and 16wk, ABR-001 Target2 8wk) or 5 mice (SpCas9 Target1 8wk and 16wk, ABR-001 Target2 16wk, ABR-001 Target1+2 8wk and 16wk). Squares and triangles represent a given mouse at 8 and 16 weeks, respectively. The input represents the CD34+ population that was engrafted, represented by a circle (n=1). b. HbF analysis in mouse bone marrow was done by intracellular flow cytometry at 8 and 16 weeks post transplantation. Bars represent the mean of n=3 (Untransfected 16wk, SpCas9 Target1 16wk, ABR-001 Target1+2 16wk), 4 (Untransfected 8wk, ABR-001 Target1+2 8wk, ABR-001 Target2 8wk and 16wk), or 5 mice (SpCas9 Target1 8wk), with each dot representing a mouse. c-d. Mouse peripheral blood (c) and bone marrow (d) was assessed for % human chimerism by flow cytometry at 8 weeks and 16 weeks post transplantation. Bars represent mean and error bars represent s.d. of n=4 (Untransfected 8wk and 16wk, ABR-001 Target2 8wk) or 5 (SpCas9 Target1 8wk and 16wk, ABR-001 Target2 16wk, ABR-001 Target1+2 8wk and 16wk) replicates, with each dot representing a mouse. e-f. Different lineage marker analysis of mouse peripheral blood (e) and bone marrow (f) by flow cytometry at 16 weeks post transplantation. Bars represent the mean. Error bars represent s.d. of n=4 replicates (Untransfected) or n=5 replicates (SpCas9 and ABR-001). Source Data are provided as a Source Data file.

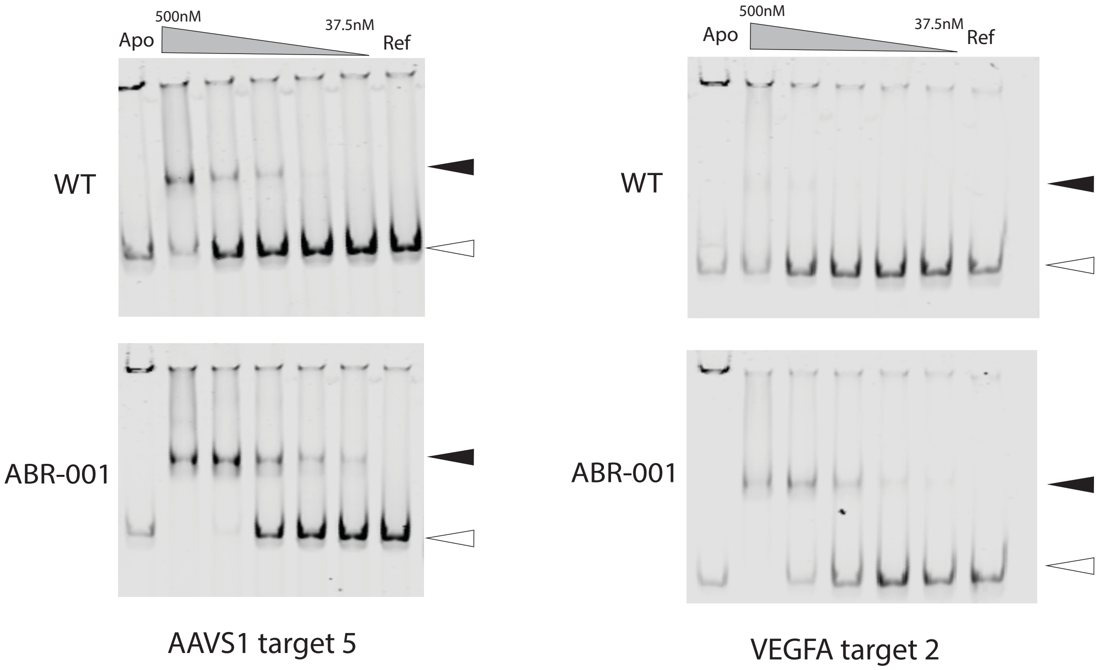

Supplementary Fig. 17. ABR-001 exhibits enhanced target DNA binding. Electrophoretic mobility shift analysis (EMSA) of Cas12i2 WT and ABR-001 binding of target dsDNA. Open arrows indicate unbound dsDNA. Black arrows indicate dsDNA bound to increasing concentrations of RNP (effector and gRNA complex). dsDNA alone is labeled as Ref, and effector protein alone as Apo. The gel is a representative image of n=2 experiments. Source Data are provided as a Source Data file.

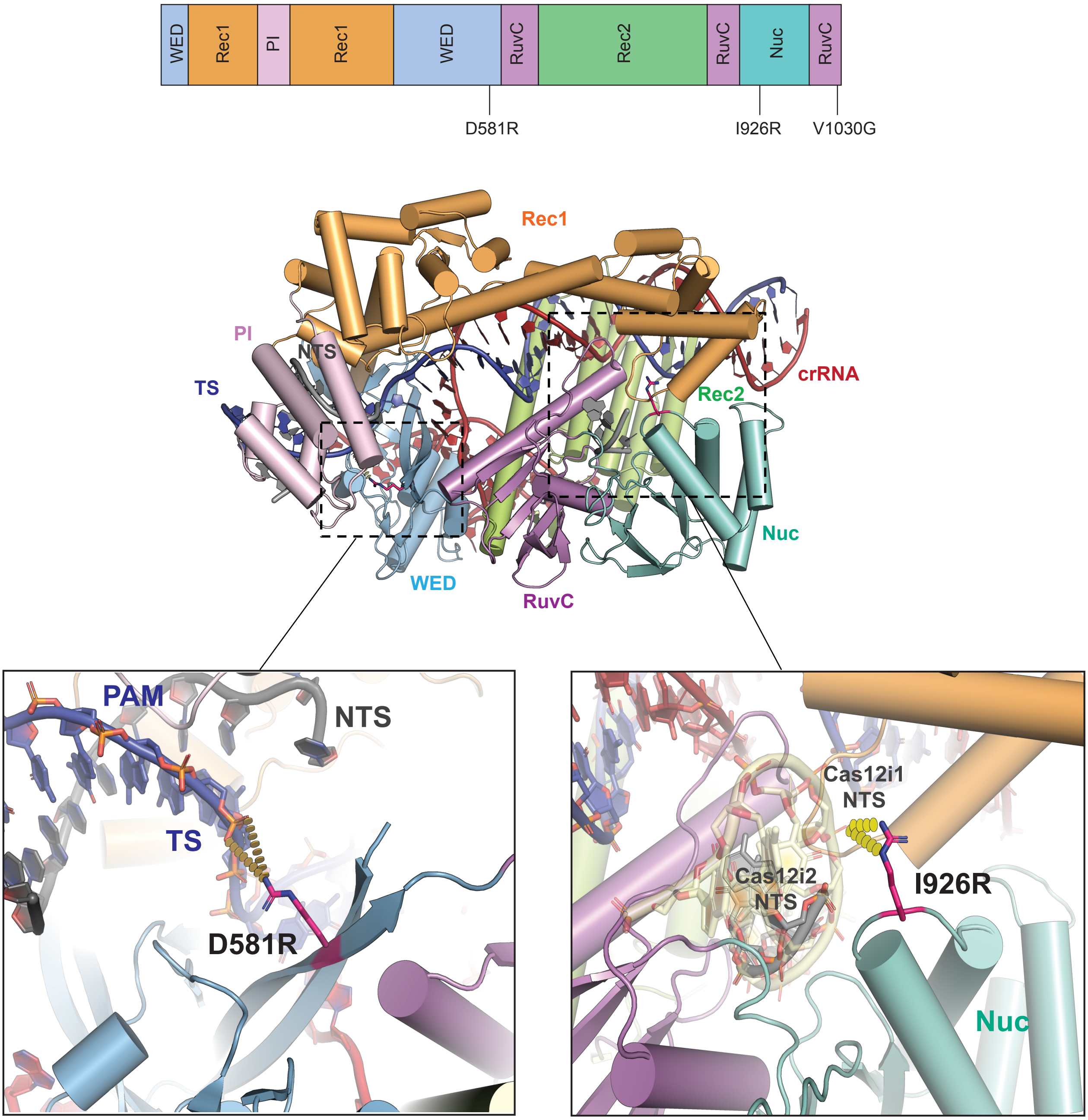

Supplementary Fig. 18. Mapping of ABR-001 indel enhancing substitutions in Cas12i2 structure. ABR-001 contains three indel enhancing substitutions, D581R, I926R and V1030G, located in WED, Nuc and WED domains, respectively. Shown are models of D581R and I926R forming electrostatic interactions with DNA backbones of the template strand (TS, D581R, left inset) and the non-template strand (NTS, I926R, right inset) based on the Cas12i2 ternary structure (PDB ID: 6LTU). Due to the lack of Cas12i2 local NTS (dark grey) structural information, a portion of Cas12i1 NTS (light grey) (PDB ID: 7EU9) is added to allow modeling of I926R interaction with NTS.

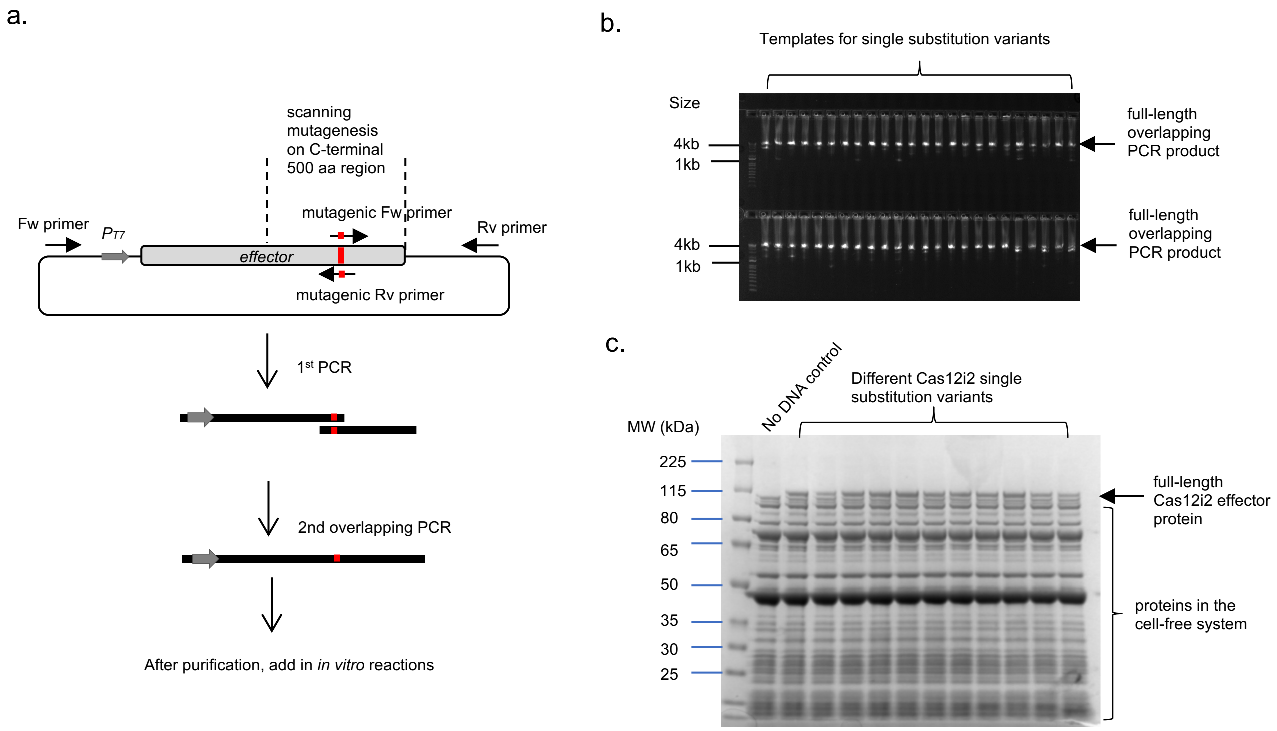

Supplementary Fig. 19. Generation of linear DNA templates for *in vitro* expression of Cas12i2 single substitution variants. a. Schematic description of a two-step overlapping PCR mutagenesis method for generating linear DNA templates for *in vitro* expression of single-substitution effector variants. b. A representative image of agarose gel showing the full-length products from the overlapping PCR method. The gel is a representative image of n=10 experiments. c. A representative image of Coomassie stained SDS-PAGE gel showing *in vitro* expression of a subset of Cas12i2 single substitution variants in the reconstituted cell-free system from linear DNA templates. The gel is a representative image of n=3 experiments. Source Data are provided as a Source Data file.

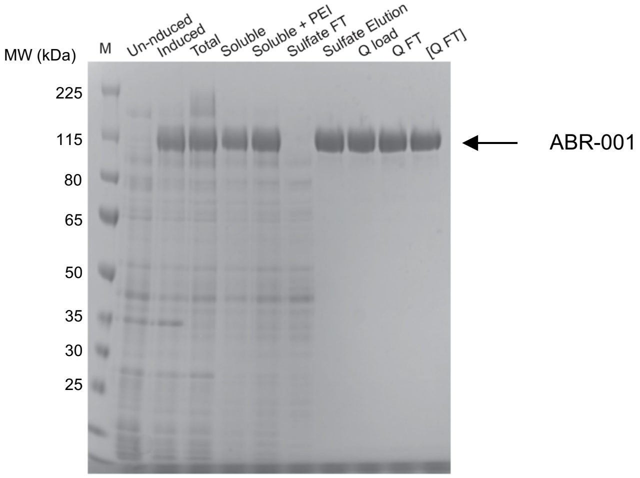

Supplementary Fig. 20. Expression and purification of ABR-001. A representative image of Coomassie stained SDS-PAGE gel showing cell lysate aliquots and column elution fractions from expression and purification experiments of ABR-001. See details in Methods. B. **.** Additional deep sequencing analysis of *in vitro* cleavage positions of ABR-001 and Cas12i2 WT on the template (TS) and non-template (NTS) strands of EMX1 target 6 and VEGFA target 5. The gel is a representative image of n=2 experiments. Source Data are provided as a Source Data file.

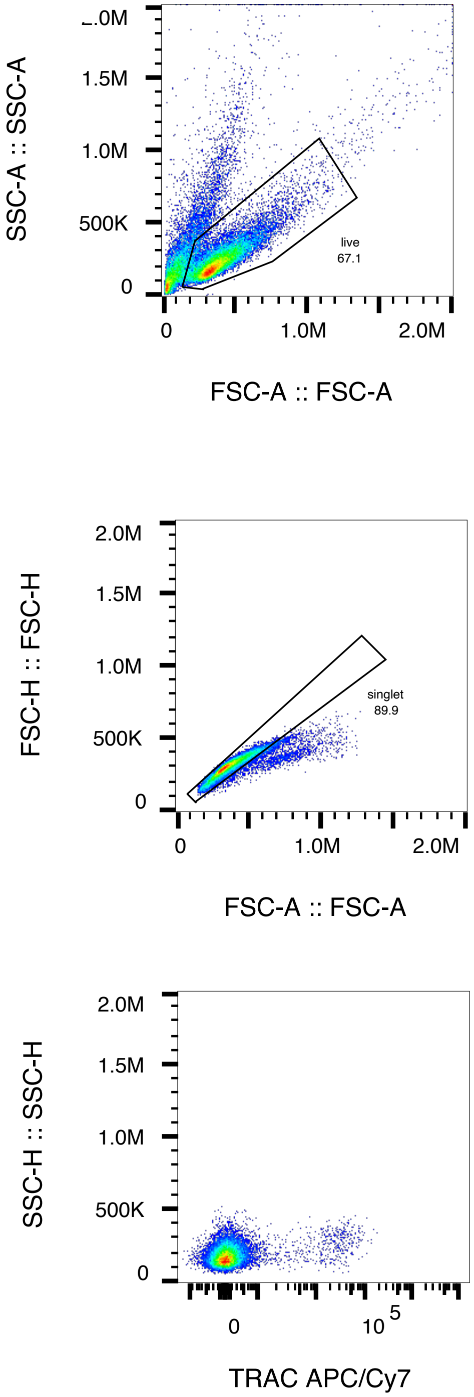

Supplementary Fig. 21. Flow cytometry gating strategy for edited CD3+ T cells.

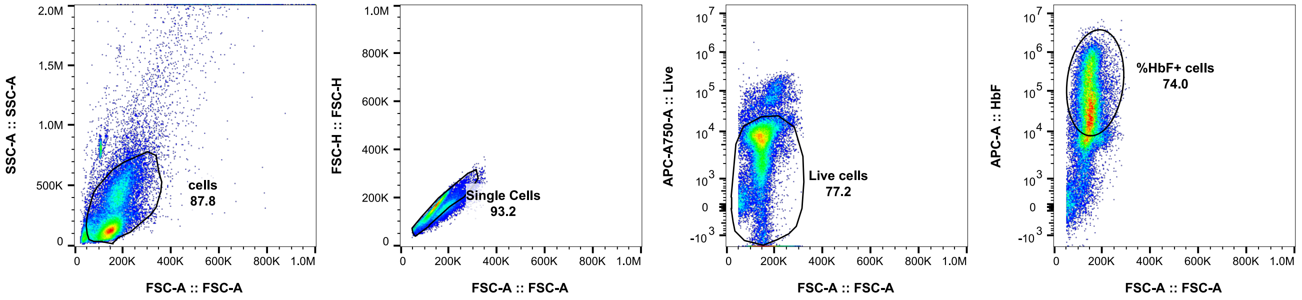

Supplementary Fig. 22. Flow cytometry gating strategy for edited CD34+ HSPCs. Plots show representative example ABR-001 Target 2 for data shown in in Fig. 3k.

Supplementary References

1. Shimizu Y*, et al.* Cell-free translation reconstituted with purified components. *Nat Biotechnol* **19**, 751-755 (2001).

2. Shimizu Y, Kanamori T, Ueda T. Protein synthesis by pure translation systems. *Methods* **36**, 299-304 (2005).

3. Jinek M, Chylinski K, Fonfara I, Hauer M, Doudna JA, Charpentier E. A programmable dual-RNA-guided DNA endonuclease in adaptive bacterial immunity. *Science* **337**, 816-821 (2012).

4. Zetsche B*, et al.* Cpf1 is a single RNA-guided endonuclease of a class 2 CRISPR-Cas system. *Cell* **163**, 759-771 (2015).

5. Strecker J*, et al.* Engineering of CRISPR-Cas12b for human genome editing. *Nat Commun* **10**, 212 (2019).

6. Liu JJ*, et al.* CasX enzymes comprise a distinct family of RNA-guided genome editors. *Nature* **566**, 218-223 (2019).

7. Harrington LB*, et al.* Programmed DNA destruction by miniature CRISPR-Cas14 enzymes. *Science* **362**, 839-842 (2018).

8. Karvelis T*, et al.* PAM recognition by miniature CRISPR-Cas12f nucleases triggers programmable double-stranded DNA target cleavage. *Nucleic Acids Res* **48**, 5016-5023 (2020).

9. Bigelyte G*, et al.* Miniature type V-F CRISPR-Cas nucleases enable targeted DNA modification in cells. *Nat Commun* **12**, 6191 (2021).

10. Pausch P*, et al.* DNA interference states of the hypercompact CRISPR-CasPhi effector. *Nat Struct Mol Biol* **28**, 652-661 (2021).

11. Pausch P*, et al.* CRISPR-CasPhi from huge phages is a hypercompact genome editor. *Science* **369**, 333-337 (2020).

12. Yan WX*, et al.* Functionally diverse type V CRISPR-Cas systems. *Science* **363**, 88-91 (2019).
