# Supplementary material for: Engineered Cas12i2 is a versatile high-efficiency platform for therapeutic genome editing
Source: Nat Commun. 2022 May 20;13:2833. doi: 10.1038/s41467-022-30465-7 (PMC9122993; doi:10.1038/s41467-022-30465-7)
Supplement: Supplementary file 3 — Reporting Summary [file 41467_2022_30465_MOESM3_ESM.pdf]

nature portfolio

Corresponding author(s): Tia DiTommaso

Last updated by author(s): 4/29/2022

Reporting Summary

Nature Portfolio wishes to improve the reproducibility of the work that we publish. This form provides structure for consistency and transparency in reporting. For further information on Nature Portfolio policies, see our Editorial Policies and the Editorial Policy Checklist.

Statistics

For all statistical analyses, confirm that the following items are present in the figure legend, table legend, main text, or Methods section.

n/a	Confirmed	
☐	☒	The exact sample size (*n*) for each experimental group/condition, given as a discrete number and unit of measurement
☐	☒	A statement on whether measurements were taken from distinct samples or whether the same sample was measured repeatedly
☐	☒	The statistical test(s) used AND whether they are one- or two-sided<br>*Only common tests should be described solely by name; describe more complex techniques in the Methods section.*
☒	☐	A description of all covariates tested
☒	☐	A description of any assumptions or corrections, such as tests of normality and adjustment for multiple comparisons
☐	☒	A full description of the statistical parameters including central tendency (e.g. means) or other basic estimates (e.g. regression coefficient) AND variation (e.g. standard deviation) or associated estimates of uncertainty (e.g. confidence intervals)
☒	☐	For null hypothesis testing, the test statistic (e.g. *F*, *t*, *r*) with confidence intervals, effect sizes, degrees of freedom and *P* value noted<br>*Give P values as exact values whenever suitable.*
☒	☐	For Bayesian analysis, information on the choice of priors and Markov chain Monte Carlo settings
☒	☐	For hierarchical and complex designs, identification of the appropriate level for tests and full reporting of outcomes
☒	☐	Estimates of effect sizes (e.g. Cohen's *d*, Pearson's *r*), indicating how they were calculated

Our web collection on statistics for biologists contains articles on many of the points above.

Software and code

Policy information about availability of computer code

Data collection	No data collection software was used.
Data analysis	Analysis of in vitro fluorescent reporter assay data is described in methods. bcl2fastq2 software version v2-20.0.422 was used in sequencing analysis. Custom code was used for indel analysis though analysis with CRISPresso will yield comparable results. Custom code was used for TTISS analysis, tho ugh analysis with BrowswerGenome.org will yield comparable results. Code will be made available upon reasonable request.FlowJo version 10.8.1 was used for flow cytometry analysis.

For manuscripts utilizing custom algorithms or software that are central to the research but not yet described in published literature, software must be made available to editors and reviewers. We strongly encourage code deposition in a community repository (e.g. GitHub). See the Nature Portfolio guidelines for submitting code & software for further information.

Data

Policy information about availability of data

All manuscripts must include a data availability statement. This statement should provide the following information, where applicable:

- Accession codes, unique identifiers, or web links for publicly available datasets
- A description of any restrictions on data availability
- For clinical datasets or third party data, please ensure that the statement adheres to our policy

Data that support the results of the present study are available within the article, the Supplementary Information file, and in the Source Data file.
Flow cytometry data is available on FLOWReposity.org under Repository IDs: FR-FCM-Z57L, FR-FCM-Z57N, FR-FCM-Z57J,FR-FCM-Z57P,FR-FCM-Z57V, FR-FCM-Z57U
All DNA sequencing data is available on the NCBI Sequence Read Archive (SRA) under the Bioproject ID PRJNA829411,
https://www.ncbi.nlm.nih.gov/sra/?term=PRJNA829411

Field-specific reporting

Please select the one below that is the best fit for your research. If you are not sure, read the appropriate sections before making your selection.

- [x] Life sciences
- [ ] Behavioural & social sciences
- [ ] Ecological, evolutionary & environmental sciences

For a reference copy of the document with all sections, see nature.com/documents/nr-reporting-summary-flat.pdf

Life sciences study design

All studies must disclose on these points even when the disclosure is negative.

Sample size

The sample size was not predefined. The engraftment study was modeled after previously published studies conducted in similar model systems with similar size groups (Chang et.al, Mol Ther Methods Clin Dev, 2017; Psatha et. al, Mol Ther Methods Clin Dev, 2018).

Data exclusions

In Supplementary Fig.13b, % Hbf in hCD45+ cells is not reported in mouse samples with insufficient cells required for flow cytometry.

Replication

All in vitro experiments were confirmed by a minimum of at least one independent replication unless otherwise noted (e.g. TTISS). The number of independent replications for each experiment is specified in the figure legends. All data points represent measurements taken from distinct samples.

The TTISS experiment was designed with controls to enable comparison with previously published work (Schmid-Burgk et al., Mol Cell, 2020; Tsai et al., Nat Biotechnology, 2015). Editing rates were confirmed to be consistent across technical replicates (i.e. individually transfected wells), as shown in figure 2b. Sensitivity, and therefore utility, for integration-dependent off-target screens is dependent upon cell inputs and sequencing depth, each of which were sufficiently achieved in this study and are in line with the current literature precedent.

Randomization

Mice were randomized be weight into different groups. No clinical research study with group randomization was involved in this study.

Blinding

No clinical research study with blinding was involved in this study.

Reporting for specific materials, systems and methods

We require information from authors about some types of materials, experimental systems and methods used in many studies. Here, indicate whether each material, system or method listed is relevant to your study. If you are not sure if a list item applies to your research, read the appropriate section before selecting a response.

Materials & experimental systems

n/a	Involved in the study
[ ]	[x] Antibodies
[ ]	[x] Eukaryotic cell lines
[x]	[ ] Palaeontology and archaeology
[ ]	[x] Animals and other organisms
[x]	[ ] Human research participants
[x]	[ ] Clinical data
[x]	[ ] Dual use research of concern

Methods

n/a	Involved in the study
[x]	[ ] ChIP-seq
[ ]	[x] Flow cytometry
[x]	[ ] MRI-based neuroimaging

Antibodies

Antibodies used

Antibodies used are described in Methods and listed here (Antibody, Clone, Fluorophore, Protein, Vendor, Catalog#, Dilution):
Anti-human B2M, 2M2, PE, Human B2M, BioLegend, 316305, 1:20
Anti-human TRAC, IP26, APC/Cy7, Human TRAC, BioLegend, 306728, 1:20
Anti-human HLA-DR/DP/DQ, Tü39, FITC, Human HLA-DR/DP/DQ, BioLegend, 361706, 1:20
Anti-human CD45, HI30, APC, Human CD45, BioLegend, 304012, 1:20
Anti-mouse CD45, 30-F11, BV785, Mouse CD45, BioLegend, 103149, 1:40
Anti-human CD15, HI98, PE, Human CD15, BioLegend, 301906, 1:20
Anti-human CD66b, G10F5, PE, Human CD66b, BioLegend, 305106, 1:20
Anti-human CD19, HIB19, BV421, Human CD19, BioLegend, 302234, 1:20
Anti-human CD20, 2H7, BV421, Human CD20, BioLegend, 302330, 1:20
Anti-human CD71, CY1G4, FITC, Human CD71, BioLegend, 334104, 1:20
Anti-human HbF, HBF-1, APC, Human HbF, Thermofisher, MHFH05, 1:20

	Anti-human HbF, HBF-1 FITC, Human HbF, Thermofisher, MHFH04, 1:20
Validation	Validation is provided on manufacturers' websites:<br>Anti-human B2M BioLegend 316305<br>https://www.biolegend.com/en-us/products/pe-anti-human-beta2-microglobulin-antibody-3080<br><br>Anti-human TRAC BioLegend 306728<br>https://www.biolegend.com/en-us/products/apc-cyanine7-anti-human-tcr-alpha-beta-antibody-12516<br><br>Anti-human HLA-DR/DP/DQ BioLegend 361706<br>https://www.biolegend.com/en-us/products/fitc-anti-human-hla-dr-dp-dq-antibody-9376<br><br>Anti-human CD45 BioLegend 304012<br>https://www.biolegend.com/en-us/products/apc-anti-human-cd45-antibody-705<br><br>Anti-human CD15 BioLegend 301906<br>https://www.biolegend.com/en-us/products/pe-anti-human-cd15-ssea-1-antibody-713<br><br>Anti-human CD66b BioLegend 305106<br>https://www.biolegend.com/en-us/products/pe-anti-human-cd66b-antibody-6529<br><br>Anti-human CD19 BioLegend 302234<br>https://www.biolegend.com/en-us/products/brilliant-violet-421-anti-human-cd19-antibody-7144<br><br>Anti-human CD20 BioLegend 302330<br>https://www.biolegend.com/en-us/products/brilliant-violet-421-anti-human-cd20-antibody-7192<br><br>Anti-human CD71 BioLegend 334104<br>https://www.biolegend.com/en-us/products/fitc-anti-human-cd71-antibody-4907<br><br>Anti-human HbF Thermofisher MHFH05<br>https://www.thermofisher.com/antibody/product/Fetal-Hemoglobin-Antibody-clone-HBF-1-Monoclonal/MHFH05<br><br>Anti-human HbF Thermofisher MHFH04<br>https://www.thermofisher.com/antibody/product/Fetal-Hemoglobin-Antibody-clone-HBF-1-Monoclonal/MHFH04

Eukaryotic cell lines

Policy information about cell lines

Cell line source(s)	HEK-293T: American Type Culture Collection (ATCC)<br>Human PBMC: Stem Cell Technologies Cat No. 70025<br>Human bone marrow CD34+cells: Lonza Cat No. 2M101C
Authentication	Human donor-derived cells were authenticated for their respective markers by flow cytometry
Mycoplasma contamination	Cells were not tested for mycoplasma
Commonly misidentified lines<br>(See ICLAC register)	No commonly misidentified cell lines were used in this study

Animals and other organisms

Policy information about studies involving animals; ARRIVE guidelines recommended for reporting animal research

Laboratory animals	The animal study described in this paper utilized female NOD.Cg-Prkdcscid Il2rgtm1Wjl/SzJ (NSG) mice (Mus musculus.).
Wild animals	n/a
Field-collected samples	n/a
Ethics oversight	This work was covered under an ethics protocol reviewed and approved by the Institutional Animal Care Committee at the University of British Columbia under protocol #A18-0276. During the study the care, housing and use of animals was performed in accordance with the Canadian Council on Animal Care Guidelines.

Note that full information on the approval of the study protocol must also be provided in the manuscript.

Flow Cytometry

Plots

Confirm that:

- [x] The axis labels state the marker and fluorochrome used (e.g. CD4-FITC).
- [x] The axis scales are clearly visible. Include numbers along axes only for bottom left plot of group (a 'group' is an analysis of identical markers).
- [x] All plots are contour plots with outliers or pseudocolor plots.
- [x] A numerical value for number of cells or percentage (with statistics) is provided.

Methodology

Sample preparation	PBMCs and T cells were harvested and washed twice with cold FBS stain buffer (BD #554656), fixed in Cytofix fixation buffer (BD 554655) for 15 min at 4°C, and washed twice with cold FBS stain buffer. Cells were stained with extracellular fluorescently-conjugated antibodies for 15 min, then washed twice with cold FBS stain buffer. For intracellular staining, cells were washed twice with cold 1X permeabilization buffer (BioLegend #421002) and incubated with intracellular fluorescently-conjugated antibodies for 15 min at room temperature, then washed twice with cold FBS stain buffer. Prior to flow cytometry analysis, stained cells were washed twice with cold 1X permeabilization buffer and resuspended in cold FBS stain buffer. A list of antibodies used in this study can be found in Supplementary Table 6.<br>For mouse peripheral blood and bone marrow cells, cells were collected post-transplantation and washed twice with FACS buffer (PBS + 2% FBS + 1 mM EDTA). Cells were resuspended in Mouse or Human Fc Blocking solution (BD) and incubated at room temperature for 10 min. Cells were incubated with fluorescently-conjugated antibodies for 30 min at 4°C, then washed twice with FBS buffer. Cells were analyzed using the CytoFLEX (Beckman Coulter).<br>For viability staining, diluted 7-AAD was added to each well and incubated for 10 min at room temperature. Flow cytometry was performed using a CytoFLEX S instrument (Beckman Coulter), and data were analyzed using FlowJo v10 (BD). A representative gating example can be found in Supplementary Fig. 21-22
Instrument	Beckman Coulter Cytoflex S
Software	Data was analyzed with FlowJo v10.8.1
Cell population abundance	At least 10,000 cells were gated for FSC-A x SSC-A. A FSC-A x FSC-H subgate was drawn to eliminate doublets.
Gating strategy	At least 10,000 cells were gated for FSC-A x SSC-A. A FSC-A x FSC-H subgate was drawn to eliminate doublets. Subgates for relevant markers were drawn depending on those specific markers being assessed in each individual experiment. Representative gating examples can be found in Supplementary Fig. 21-22.

- [x] Tick this box to confirm that a figure exemplifying the gating strategy is provided in the Supplementary Information.
